# A_2B_ adenosine receptor antagonists rescue lymphocyte activity in adenosine-producing patient-derived cancer models

**DOI:** 10.1136/jitc-2022-004592

**Published:** 2022-05-17

**Authors:** Apple Hui Min Tay, Rubén Prieto-Díaz, Shiyong Neo, Le Tong, Xinsong Chen, Valentina Carannante, Björn Önfelt, Johan Hartman, Felix Haglund, Maria Majellaro, Jhonny Azuaje, Xerardo Garcia-Mera, Jose M Brea, Maria I Loza, Willem Jespers, Hugo Gutierrez-de-Teran, Eddy Sotelo, Andreas Lundqvist

**Affiliations:** 1 Department of Biological Science, Nanyang Technological University, Singapore; 2 Department of Oncology-Pathology, Karolinska Institute, Stockholm, Sweden; 3 Center for Research in Biological Chemistry and Molecular Materials, University of Santiago de Compostela, Santiago de Compostela, Galicia, Spain; 4 Singapore Immunology Network SIgN, Agency for Science, Technology and Research, Singapore, Republic of Singapore; 5 Department of Microbiology Tumor and Cell Biology, Karolinska Institutet, Stockholm, Sweden; 6 Department of Applied Physics, Science for Life Laboratory, KTH Royal Institute of Technology, Stockholm, Sweden; 7 Department of Clinical Pathology and Cancer Diagnostics, Karolinska University Hospital, Stockholm, Sweden; 8 Center for Research in Molecular Medicine and Chronic Diseases, University of Santiago de Compostela, Santiago de Compostela, Galicia, Spain; 9 Department of Cell and Molecular Biology, Uppsala University, Uppsala, Sweden; 10 Department of Cell and Molecular Biology, Science for Life Laboratory, Uppsala University, Uppsala, Sweden

**Keywords:** adenosine, immunotherapy, lymphocyte activation, lymphocytes, tumor-infiltrating

## Abstract

**Background:**

Adenosine is a metabolite that suppresses antitumor immune response of T and NK cells via extracellular binding to the two subtypes of adenosine-2 receptors, A_2_ARs. While blockade of the A_2A_ARs subtype effectively rescues lymphocyte activity, with four A_2A_AR antagonists currently in anticancer clinical trials, less is known for the therapeutic potential of the other A_2B_AR blockade within cancer immunotherapy. Recent studies suggest the formation of A_2A_AR/A_2B_AR dimers in tissues that coexpress the two receptor subtypes, where the A_2B_AR plays a dominant role, suggesting it as a promising target for cancer immunotherapy.

**Methods:**

We report the synthesis and functional evaluation of five potent A_2B_AR antagonists and a dual A_2A_AR/A_2B_AR antagonist. The compounds were designed using previous pharmacological data assisted by modeling studies. Synthesis was developed using multicomponent approaches. Flow cytometry was used to evaluate the phenotype of T and NK cells on A_2B_AR antagonist treatment. Functional activity of T and NK cells was tested in patient-derived tumor spheroid models.

**Results:**

We provide data for six novel small molecules: five A_2B_AR selective antagonists and a dual A_2A_AR/A_2B_AR antagonist. The growth of patient-derived breast cancer spheroids is prevented when treated with A_2B_AR antagonists. To elucidate if this depends on increased lymphocyte activity, immune cells proliferation, and cytokine production, lymphocyte infiltration was evaluated and compared with the potent A_2A_AR antagonist AZD-4635. We find that A_2B_AR antagonists rescue T and NK cell proliferation, IFNγ and perforin production, and increase tumor infiltrating lymphocytes infiltration into tumor spheroids without altering the expression of adhesion molecules.

**Conclusions:**

Our results demonstrate that A_2B_AR is a promising target in immunotherapy, identifying ISAM-R56A as the most potent candidate for A_2B_AR blockade. Inhibition of A_2B_AR signaling restores T cell function and proliferation. Furthermore, A_2B_AR and dual A_2A_AR/A_2B_AR antagonists showed similar or better results than A_2A_AR antagonist AZD-4635 reinforcing the idea of dominant role of the A_2B_AR in the regulation of the immune system.

**WHAT IS ALREADY KNOWN ON THIS TOPIC**

- A_2B_AR is a low affinity adenosine receptor that is activated by high adenosine concentration. With adenosine being an anti-inflammatory and immunosuppressive metabolite that accumulates at high concentrations in the tumor microenvironment, targeting A_2B_AR is a promising metabolic immune checkpoint.

**WHAT THIS STUDY ADDS**

- We synthesized high affinity non-xanthine A_2B_AR antagonists with improved T and NK cell activities under exogenous and endogenous adenosine suppression. A_2B_AR inhibition furthermore improves infiltration of tumor-infiltrating lymphocytes into patient-derived 3D spheroids.

**HOW THIS STUDY MIGHT AFFECT RESEARCH, PRACTICE AND/OR POLICY**

- These findings provide practical readouts to study adenosine-mediated suppression and identified putative A_2B_AR antagonists to alleviate suppression of T and NK cells.

## Background

Accounting for its heterogeneity with more than 100 distinct types, cancer is a complex and dynamic disease.^1^ This complexity has been rationalized to 10 transforming hallmarks, of which avoiding immune destruction explains how the immune system plays an essential role during tumorigenesis.^1^ Therapies based on activating the immune system can result in beneficial responses in patients with metastatic cancer.^2^ Treatment with antibodies targeting the immunological checkpoint axis PD-1–PD-L1/2 can result in potent antitumor T cell activation and clinically meaningful long-lasting responses.^2^ Primary resistance to immune checkpoint therapy can be attributed to the absence of intratumoral T cells. This has fueled the search for strategies to convert immune-excluded tumors to immune-infiltrated tumors.^2^ Since the tumor microenvironment (TME) often imposes metabolic stress and dysregulation on tumor infiltrating lymphocytes (TILs), targeting immune metabolism represents a promising direction.^3^

Purinergic signaling involves extracellular purine nucleosides [Adenosine (ADO)] and nucleotides (ATP and AMP) as signaling molecules. ADO, an ubiquitous metabolite with critical anti-inflammatory and immunosuppressive roles, downregulates inflammatory cytokine secretion as well as decreases the effector function and proliferation of T and natural killer (NK) cells (online supplemental figure 1).^4^ Several tumors display transformed purine metabolism thereby facilitating the production of ADO and reducing its degradation.^5^ Oxygen deprivation reduces the availability of energy source and promotes the accumulation of extracellular ATP.^6^ Moreover, hypoxia is a strong inducer of the ectonucleotidases CD39 and CD73.^4^ The released ATP suffers the consecutive action of these ectonucleotidases resulting in increased ADO concentration from nanomolar (nM) in physiological conditions to micromolar (μΜ) range in the TME.^7–10^

10.1136/jitc-2022-004592.supp1 Supplementary data

Extracellular ADO binds to purinergic type-1 G-protein coupled receptors (GPCRs), accordingly named adenosine receptors (AR)—A_1_AR, A_2A_AR, A_2B_AR and A_3_AR.^4 11^ A_2A_AR is highly expressed in most immune cells, with evidences supporting that its activation in the TME suppresses antitumor immune responses,^12 13^ enhancement of regulatory T cells immune suppressive activity^14^ and inhibition of antigen presentation by dendritic cells.^15^ These reports set the foundation for inhibiting A_2A_AR in hypoxic tumors to improve antitumor immune responses. Consequently, A_2A_AR antagonism has emerged as a prototypical approach of small molecule immunotherapeutic, with recent encouraging clinical outcomes in treatment-refractory cancer,^16 17^ along the four A_2A_AR antagonists already in clinical trials.^18^

While the A_1_AR, A_2A_AR and A_3_AR receptor subtypes bind ADO with high affinity, the A_2B_AR exhibits a low affinity profile. Thus, A_2B_AR will only be activated by high ADO concentrations which usually takes place under extreme environmental cues like inflammation, injury, hypoxia or cellular stress.^19 20^ Recent evidence suggests A_2B_AR roles in cancer,^5 21^ following early studies highlighting its activation as a promoter for tumor proliferation,^5 6^ angiogenesis,^22^ cell invasion and metastasis.^5^ Furthermore, the presence of A_2B_AR in mast cells, neutrophils, dendritic cells, macrophages and lymphocytes has shown important immunoregulatory roles within the immune suppressive TME.^4 5 23^ The expression and signaling of the two A_2_AR subtypes are highly affected by pathological conditions, with the A_2B_AR/A_2A_AR expression ratio rapidly increasing under hypoxic conditions.^5 19 24^ In this context, a recent study demonstrated extensive heteromeric complex formation in tissues where A_2B_ and A_2A_ ARs were coexpressed.^19^ A_2A_AR was previously shown to be involved in regulation of A_2B_AR cell surface expression.^25^ Moreover, a dramatically altered pharmacology of the A_2A_AR was observed when coexpressed with the A_2B_AR with selective A_2A_AR ligands loosing high affinity binding to A_2A_AR and showing reduced potency. These would have major implications for the clinical use of A_2A_AR ligands, as they would fail to modulate the receptor in an A_2A_AR-A_2B_AR heterodimer context. Instead, the A_2A_AR-A_2B_AR heterodimer and the A_2B_AR could be considered as novel promising pharmacological targets for cancer immunotherapy.

In this study, six non-xanthinic A_2_AR antagonists, five selective A_2B_AR and a dual A_2A_AR/A_2B_AR, were evaluated for their immunomodulatory effect. Non-xanthinic scaffold exhibits improved pharmacokinetic properties and bring structural novelty to adenosine antagonists reported to date.^26 27^ Using healthy donor in vitro and patient-derived ex vivo models, we demonstrated that antagonizing A_2B_AR signaling significantly alleviated adenosine-mediated suppression across different lymphocyte subsets. Notably, marked differences in the outcomes of A_2B_AR antagonist drug screening were observed when comparing exogenous and endogenously produced ADO. These results show a comparative immunological footprint among different A_2B_AR antagonists, a dual A_2B_AR/A_2A_AR antagonist and an A_2A_AR antagonist in clinical trials, strongly suggesting that the A_2B_AR is a promising target in cancer immunotherapy.

## Materials and methods

### Chemistry

The synthesis of the previously described antagonists, analytical procedure and spectroscopic and analytical data for all the compounds is detailed in online supplemental information. For the synthesis of ISAM–R56A, a mixture of isopropyl 4-(furan-2-yl)−2-methyl-1,4-dihydrobenzo^4 5^ imidazo[1,2 a]pyrimidine-3-carboxylate (ISAM–140) (1 mmol), 2-fluorobenzyl bromide (3 mmol) and potassium carbonate (4 mmol) in 4 mL of DMF was orbitally stirred in a coated Kimble vial at 80°C for 5 hours. After completion of the reaction, as indicated by TLC, the solvent was removed in vacuum and the obtained oily residue was purified by column chromatography on silica gel to obtain two regioisomers (ISAM–R56A and ISAM–R56B). For the synthesis of ISAM–M89A, a mixture of 2-amino-5-chlorobenzimidazole (7.5 mmol), 3-furanecarboxaldehyde (5 mmol), isopropyl acetoacetate (5 mmol) and ZnCl_2_ (0.5 mmol) in 2.5 mL of THF was orbitally stirred in a coated Kimble vial at 80°C for 12 hours. After completion of the reaction, as indicated by TLC, the solvent was removed and the obtained oily residue was purified by column chromatography on silica gel, to obtain two regioisomers (ISAM–M89A and ISAM–M89B).

10.1136/jitc-2022-004592.supp2 Supplementary data

### Binding affinity of adenosine receptor subtypes

The affinity and selectivity profiles of the ligands obtained was studied in vitro, radioligand binding assays, at the four human specific ARs subtypes, using experimental protocols previously described.^28–30^ All ligands were prepared and tested as racemic mixtures. Human ARs expressed in transfected CHO (A_1_AR), HeLa (A_2A_AR and A_3_AR) and HEK-293 (A_2B_AR) cells were employed. The following radioligands were used for binding experiments: [^3^H]DPCPX for A_1_AR and A_2B_AR, at 2 and 25 nM, respectively; [^3^H]ZM241385 at 3 nM for A_2A_AR; and [^3^H]NECA at 30 nM for A_3_AR. Non-specific binding was determined in the presence of R-PIA 10 µM for A_1_AR, NECA 50 µM for A_2A_AR, NECA 400 μΜ for A_2B_AR and R-PIA 100 μΜ for A_3_AR. The biological data are expressed as K*_i_* (nM, n=3. K*_i_* values were obtained by fitting the data with non-linear regression using Prism 5.0 software (GraphPad, San Diego, CA, USA). Results are the mean of three experiments, each performed in duplicate.

### Blockade of *h*CD73 assays

The selected AR antagonists were tested at 1 µM and 10 µM. Experiments (human CD73) were carried out in a white 384-Optiplate (Perkin Elmer 6007290). Test compounds and the standard (α-β-methylene adenosine, Sigma M3763), 0.5 µg/mL enzyme (Cayman RYD-5795-EN-010), 300 µM AMP (Sigma A2252) and 100 µM ATP (A2383) were added in a final volume of 25 µL/well, using 25 mM Tris-HCl, 5 mM MgCl_2_·6H_2_O, pH=7.4 as assay buffer. The reaction mixture was incubated at 37°C for 15 min, after incubation 25 µL of Cell Titer-Glo Luminescent cell viability (Promega G7571) was added and shaken during 2 min before incubation at RT for 10 min. Luminescence at 100 ms was measured in the Perkin Elmer Enspire multimode plate reader.

### cAMP assays

Assays were performed in transfected A_2B_AR using a cyclic AMP (cAMP) enzyme immunoassay kit (Amersham Biosciences) following previously described protocols.^28 30^ HEK-293 cells were seeded (10,000 cells/well) in 96-well culture plates and incubated at 37°C in an atmosphere with 5% CO_2_ in Eagle’s Medium Nutrient Mixture F-12 (EMEM F-12), containing 10% fetal calf serum (FCS) and 1% L-Glutamine. Cells were washed three times with 200 µL assay medium (EMEM-F12 and 25 mM HEPES pH=7.4) and pre-incubated with assay medium containing 30 µM rolipram and test compounds at 37°C for 15 min. Ten µM NECA was incubated for 15 min at 37°C (total incubation time 30 min). Reaction was stopped with lysis buffer supplied in the kit and the enzyme immunoassay was carried out for detection of intracellular cAMP at 450 nm in an Ultra Evolution detector (Tecan). The dose-response curve of NECA-elicited cAMP formation was used to determine the initial choice of A_2B_AR antagonist concentration (online supplemental figure 2).

### Computational modeling

**Figure 1:**
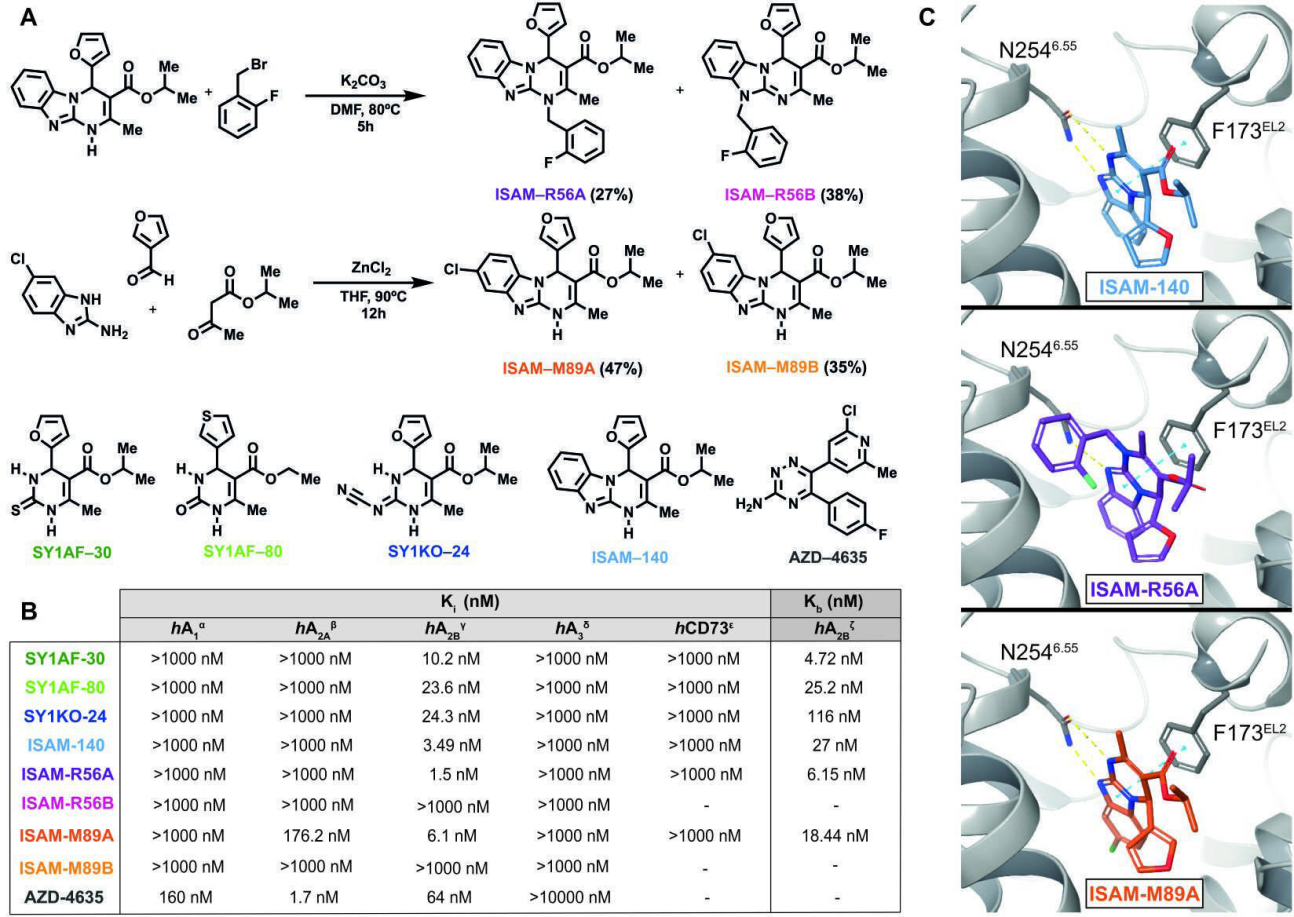
Pyrimidine derivatives as potent and highly selective A_2B_AR antagonists. (A) Synthesis of the novel A_2B_AR antagonist (ISAM–R56A) and the dual A_2A_AR/A_2B_AR antagonist (ISAM–M89A) and structure of four A_2B_AR antagonists previously published and the A_2A_AR clinical candidate (AZD-4635) employed in the study.^17 26–28^ (B) Adenosine receptors binding data, A_2B_AR functional data and *h*CD73 inhibitory data of the A_2B_AR antagonists employed in the study. ^α)^Displacement of specific [^3^H]DPCPX binding in human CHO cells expressed as *K*_i_ in nM (n=3). ^β)^Displacement of specific [^3^H]4-(2-[7-amino-2-(2-furyl)^1 2 4^ triazolo[2,3 a]^1 3 5^ triazin-5-ylamino]ethyl)phenol binding in human HeLa cells expressed as *K*_i_ in nM (n=3). ^γ)^Displacement of specific [^3^H]DPCPX binding in human HEK-293 cells expressed as *K*_i_ in nM (n=3). ^δ)^Displacement of specific [^3^H]NECA binding in human HeLa cells expressed as *K*_i_ in nM (n=3). ^ε)^Inhibition of *h*CD73 in presence of α-β-methylene adenosine, ATP and AMP (expressed as *K*_i_ in nM). ^ζ)^cAMP production in HEK-293 cells in presence of NECA and test compound at a concentration of 10 µM expressed as K_b_ in nM (n=3) (concentration-response curves shown in online supplemental figure 1). (C) Binding mode of the three most potent compounds disclosed in this study. All three compounds interact with N254^6.55^ and F173^EL2^, both of which are crucial in AR ligand recognition. Up: ISAM–140 (light blue), center: ISAM–R56A (purple) only forms one hydrogen bond with N254^6.55^, but additionally explores a pocket between TM6 and TM7, down: ISAM–M89A (orange), where the Cl atom (green stick) protrudes deep in the binding pocket.

A previously reported model of the A_2B_AR in complex with the reference antagonist ISAM–140 was the starting point to investigate the binding mode of the antagonists here reported.^31^ Briefly, the inactive hA_2B_AR conformation was generated by homology modeling based on curated alignment with the A_2A_AR, of known structure, followed by some refinement steps as previously described.^28 32^ The initial binding orientation of ISAM–140, initially obtained by automated docking with GOLD,^30^ was herein refined with a round of MD simulations, consisting of: (i) insertion of the A_2B_AR-ISAM–140 complex on an atomistic model of the membrane, solvation, and a 5 ns MD equilibration protocol as implemented in the PyMemDyn module of the GPCR-ModSim webserver;^33^ (ii) a short MD equilibration of the binding site, consisting on a 25 Å radius solvated sphere with the software Q^34^ as detailed in online supplemental information. The remaining ligands were modeled in the equivalent stereoisomer and aligned with the ‘Flexible Ligand Superposition’ in Schrödinger^35^ to this pose of ISAM–140. Each complex was subject to the same MD equilibration of the binding site outlined for ISAM–140, with representative snapshots shown in figure 1C.

### PBMC and immune cell isolation

Peripheral blood mononuclear cells (PBMCs) were isolated from healthy blood donors’ buffy coat and patient’s blood after Ficoll density gradient centrifugation (GE Healthcare). NK cells were isolated using MACS MicroBead Human NK cell isolation kit (Miltenyi Biotec).

### Tumor tissue processing and cell lines

Fresh tumor tissue resections were digested and processed into single cell suspension using gentleMACS tumor dissociation kit (Miltenyi Biotec). Tumor cells were then isolated by negative selection using a tumor cell isolation kit (Miltenyi Biotec). Adherent cells were passaged at least five times before being used for experiments. All cell lines were maintained in RPMI 1640 or DMEM GlutaMAX media (Thermo Fisher Scientific) supplemented with 10% HyClone fetal bovine serum (FBS) (GE Healthcare) and 1% Penicillin Streptomycin (PS) (Life Technologies). A tumor cell line and TIL culture from a breast cancer specimen was established as previously described.^36^ Human osteosarcoma cell line U2OS (ATCC) was used in comparison to patient-derived sarcoma cell lines for relative adenosine production and ADO ectonucleotidase expression by flow cytometry.

### Expansion of tumor infiltrating lymphocytes

Digested tumor resections were cultured in suspension with 5% human serum in AIM-V (Thermo Fisher Scientific) or X-VIVO20 (Lonza) media supplemented with 3000 IU/mL of IL-15 (Novartis). After 7 days, irradiated PBMCs (100 Gy) pooled from at least three healthy donors were added as feeder cells at 200:1 ratio with addition of anti-CD3 functional grade antibody and 500 IU/mL of IL-15. After an additional 10 days of culture, TILs were harvested and analyzed for purity using flow cytometry and maintained with 500IU/mL of IL-15.

### Cell viability assay (Cell-Titer Glo)

From breast cancer single cell suspension, 3×10^3^ cells were seeded and cultured in an ultra-low attachment 384-well plate (Corning) with DMEM-F12 media (Thermo Fisher Scientific) containing 20% FBS and 1% PS. Treatment with A_2B_AR antagonist at various concentrations are stated in the Results section. After 4 days, Cell-Titer Glo reagent (Promega) was used to measure cell viability in accordance to the manufacturer’s instruction. EnSpire Multilabel Reader (PerkinElmer) was used to read the luminescence.

### Proliferation assay with exogenous adenosine

Freshly isolated PBMCs and NK cells were stained with 5 µM FITC-conjugated carboxyfluorescein succinimidyl ester (CFSE, BioLegend) in phosphate-buffered saline (PBS) (Life Technologies) at room temperature for 5 min. CSFE stained cells were washed with flow cytometer buffer three times. CSFE-labeled healthy PBMCs and NK cells were cultured in 96-well plates with X-VIVO20% and 1% PS for 3 and 6 days, respectively. Healthy PBMCs were incubated with Human T-activator CD3/CD28 beads at 1:4 ratio (Thermo Fisher Scientific) and IL-2 (Novartis) at 100 IU/mL. Healthy donor NK cells were incubated with 1000 IU/mL IL-2 only. 2×10^5^ PBMCs and 5×10^4^ NK cells were counted for treatment with ADO (Sigma-Aldrich) and 12 μM of A_2B_AR antagonist at the same day.

### Proliferation assay with CD73-expressing patient-derived sarcoma spheroid

A malignant peripheral nerve sheath tumor (MPNST) patient-derived sarcoma cell line was used to grow spheroid by seeding 1×10^4^ cells per well in a 96-well ultra-low attachment plate (Thermo Fisher Scientific) with DMEM-F12 media containing 20% FBS and 1% PS for 5 days. 1×10^5^ CSFE-labeled healthy PBMCs, ratio of 10:1, were added to the CD73 expressing, adenosine-producing spheroid at day five. Similar to the proliferation assay with exogenous ADO, CD3/CD28 beads at 1:4 ratio and IL-2 at 100 IU/mL were added. A_2B_AR antagonism treatment at 12 μM was added at the same day.

### Spheroid infiltration by autologous TILs

Spheroids were prepared using patient-derived sarcoma cell lines. 1×10^4^ cells were seeded per well in 96-well ultra-low attachment plate with DMEM-F12 media containing 20% FBS and 1% PS for 5 days. 3×10^4^ CSFE-labeled autologous expanded TILs, at Effector:target ratio of 3:1, were added to the spheroid at day five with A_2B_AR antagonism treatment at 12 µM. After 3 days, spheroid was removed and split into two groups—IN and OUT. IN indicates TILs infiltrated into the sphere, while OUT indicates TILs that did not infiltrate into the sphere. Spheroids were washed with PBS at least two times. GentleMACS tumor dissociation kit (Miltenyi Biotec) was used to digest the spheroids for FC analysis.

### Real-time imaging

Brightfield and phase contrast images under 4X objective were acquired every 6 hours on IncuCyte S3 system (Essen BioScience). For breast cancer patient-derived spheroid culture, 1×10^4^ cells per well were seeded with A_2B_AR antagonist at 12 µM treatment for 6 days real time imaging. For spheroid infiltration, after the addition of CSFE-labeled autologous expanded TILs and treatment with A_2B_AR antagonist at 12 µM, green fluorescence images were acquired for 3 days. All spheroid invasion analysis was performed using top hat segmentation with IncuCyte software.

### Extracellular ADO uptake assay via pAMPK staining

Experimental setup was adopted from a previous study whereby AR antagonist was added to pretreat cells before exogenous adenosine treatment for intracellular and cytokine staining.^37^ In brief, PBMCs were treated with 12 µM of A_2B_AR antagonists for 90 min before the addition of 50 µM of adenosine for 2 hours. Cells were harvested after 3 hours of CD3/CD28 bead stimulation before intracellular staining with phosphor-AMPK (Thr183, Thr172) rabbit primary antibody (Thermo Fisher Scientific) and antirabbit secondary antibody (BD biosciences).

### IFNy and perforin cytokine production assay

Similar experimental setup was performed as the extracellular ADO uptake assay. Cells were harvested after 2 days of CD3/CD28 bead stimulation before treatment with PMA/ionomycin (Sigma Aldrich) and golgi-inhibitors (BD biosciences). After 3 hours of subsequent incubation, intracellular staining was performed for IFNy and perforin (online supplemental table 1).

### Flow cytometry analysis

Cell surface was stained with mouse monoclonal antihuman antibodies against CD3, CD4, CD8, CD45RA, CD56 and CD19 listed in online supplemental table 1. Cell surface antibodies and live/dead (L/D) marker were incubated with samples at 4°C for 20 min after washing two times with flow cytometry buffer containing 5% FBS in PBS. For spheroid infiltrated TIL phenotyping, digested IN and OUT spheroids were stained with cell surface marker and analyzed on NovoCyte (ACEA Bioscience) with the use of FlowJo software (Tree Star) by gating single cell based on forward and side scatters. A representative gating strategy for CD8 naïve T cells is shown in online supplemental figure 3A. Compensated flow cytometry standard (FCS) files with only live cells were concatenated for downstream tSNE analysis using the ‘cytofkit’ R package (https://github.com/JinmiaoChenLab/cytofkit).

### Statistical analysis

Experimental replicates are presented as mean±SD and median in box plot stated in the figure legend of the result section. Statistical analysis was performed using Prism 8 (GraphPad Software) and stated in figure legends.

## Results

### Synthesis and pharmacological characterization of potent A_2B_AR antagonists

The pyrimidine derivatives studied here were obtained using a modified procedure of the reliable Biginelli reaction,^38^ consisting of the catalyzed condensation of an aldehyde, a β-keto-ester and a 1,3-dinucleophile. The synthesis and binding data of SY1AF–30,^29^ SY1AF–80,^29^ SY1KO-24^28^ and ISAM-140^30^ (figure 1A) were recently described by our group in the context of a program to develop novel A_2B_AR antagonists. In addition, we present two previously undisclosed derivatives (ISAM–R56A and ISAM–R89A, figure 1A). These compounds are structurally related to ISAM–140 and they were discovered in the context of the structure activity relationship (SAR) exploration and structural diversification of the tricyclic scaffold present in this prototypical A_2B_AR antagonist. Besides providing distinctive and not-self-evident structural novelties, this pair of compounds offer interesting pharmacological data from quantitative (K*_i_* in the low nM range) and qualitative (dual profile) points of view.

The synthetic pathway employed to prepare the novel A_2B_AR ligands is shown in figure 1A, where it can be observed that both transformations exploit the tautomerism present in the precursors. Briefly, treatment of ISAM–140 with 2-fluorobenzyl bromide under basic conditions produced a mixture of regioisomers (ISAM–R56A and ISAM–R56B) that was separated using column chromatography. The Biginelli-inspired ZnCl_2_-catalyzed condensation of 2-amino-5-chlorobenzimidazole, 3-furanecarboxaldehyde and isopropyl acetoacetate gave a (1:1) mixture of two tricyclic regioisomers (ISAM–M89A and ISAM–M89B) that differ in the position (7/8) of the halogen atom. The unequivocal assignation for each regioisomer was determined by NMR techniques.

The binding data obtained from the five A_2B_AR antagonists, the dual A_2B_AR/A_2A_AR ligand and the A_2A_AR clinical candidate (AZD-4635) are depicted in figure 1B. The previously reported A_2B_AR antagonists (SY1AF–30, SY1AF–80, SY1KO–24, ISAM–140) exhibit affinity values in the low nanomolar range (K*_i_* =3.50–24.3 nM) and excellent subtype selectivity. Moreover, the diverse substitution pattern at the central pyrimidine scaffold provides chemical entities with different topologies, physicochemical features and distinctive binding modes, lately affecting its pharmacodynamic and pharmacokinetic profiles. ISAM–M89A and ISAM–R56A are novel non-xanthinic A_2B_AR ligands discovered during the detailed exploration of the SAR around the tricyclic scaffold of ISAM–140 by halogen introduction and *N*-alkylation, respectively.

The selected A_2B_AR and A_2B_AR/A_2A_AR ligands were tested in cAMP assays (figure 1B) to evaluate their ability to inhibit NECA-stimulated (100 nM) cAMP production. These experiments demonstrated that all of them inhibited cAMP accumulation, thus validating its A_2B_AR antagonistic behavior (figure 1B). A comparison of their K*_i_* and K_B_ values revealed complete agreement (data within 1–7-fold) between the binding and functional assays. Ectonucleotidases, in particular CD73, play a key role in the context of adenosine-mediated tumor immune escape.^4^ Consequently, we evaluated the effect of A_2B_AR ligands on the blockade of *h*CD73 as part of the pharmacological characterization of the selected A_2B_AR ligands (figure 1B). These experiments revealed that none of the five A_2B_AR antagonists, nor the A_2A_/A_2B_ dual antagonist, showed any noticeable inhibitory effect on *h*CD73 (1 µM or 10 µM). These data excluded a dual A_2B_AR-CD73 inhibition, allowing us to attribute the pharmacological effects described below to the specific A_2B_AR antagonistic effect (or the dual A_2A_/A_2B_ antagonism in the case of ISAM–M89A) of these ligands.

### Binding mode of A_2B_AR antagonists

The binding mode of the three most potent compounds (ISAM–140, ISAM–R56A and ISAM–M89A) was investigated using a previously reported A_2B_AR homology model in complex with ISAM–140.^31^ Such a model could successfully explain the stereospecific recognition of trifluorinated derivatives (figure 1C), and the new antagonists ISAM–R56A and ISAM–M89A could indeed adopt an analogous binding mode within the orthosteric A_2B_AR cavity (figure 1C). In all three cases, the tricyclic core is stabilized by interaction with the AR conserved residues N254^6.55^ and F173^EL2^, a common denominator for AR ligands.^39^ Ligand ISAM–M89A specifically maintained a double hydrogen bond with N254^6.55^, allowing the chlorine atom in position eight to bind deeper in the binding pocket, surrounded by H251^6.52^, N186^5.42^ and Q90^3.37^. The N_1_ substituted ISAM–R56A, which could only retain one hydrogen bond with N254^6.55^, presented, on the other hand, the extensive interactions of the 2-fluorobenzyl group in a A_2B_AR specific hydrophobic pocket located between TM6 and TM7, formed by residues V253(I)^6.54^, V250(L)^6.51^, A271(L)^7.34^, M272^7.35^, A275^7.38^ and I276^7.39^ (parenthesis indicating the corresponding A_2A_AR residues, if different). These specific interactions might explain the high affinity and the retained high selective profile for the A_2B_AR.

### Blockade of A_2B_AR reduces patient-derived breast cancer spheroid growth

**Figure 2:**
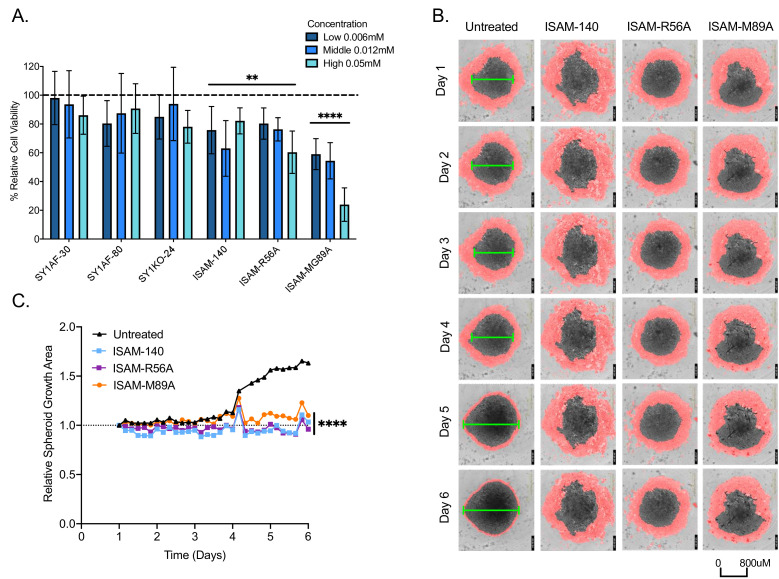
Antitumor effect of A_2B_AR antagonists on breast cancer patient-derived spheroid cultures. (A) Relative cell viability to untreated antagonist control spheroid cultures (n=3, mean±SD) measured by Cell-Titer Glo assay. (B) Representative real time imaging of spheroid cultures over 6 days under 4X objective, with untreated (first column) or added 12 µM concentration respective of A_2B_AR antagonists indicated. Red mask demarcates area with cells surrounding the spheroid. Green line indicates estimated diameter of spheroid body. (C) Relative area of the representative spheroid growth (as shown in B) normalized to day 1. Statistical analysis—2-way (A) and 1-way (C) ANOVA with Dunnett’s multiple comparisons to untreated antagonist control was performed with *p<0.05, **p<0.01, ***p<0.001, and ****p<0.0001.

The expression of A_2B_AR gene (*ADORA2B*) was investigated using *The Cancer Genome Atlas (TCGA*)—Pan cancer publicly available dataset across 33 tumor types (online supplemental figure 4A–D). With high ADORA2B gene expression in breast cancer, the blockade of the adenosinergic pathway has been widely studied in breast cancer.^40–49^ To investigate if our novel A_2B_AR antagonists would affect breast cancer growth, we used a drug screening platform based on breast tumor resections.^50^ It is composed of majority tumor cells at 73.2%, with 11.2% immune cells based on flow cytometry analysis of EpCAM and CD45 expression, respectively (online supplemental figure 4E,F). On exposure to ISAM–140, ISAM–R56A and ISAM–M89A for 4 days, the relative cell viability of these breast cancer spheroids was significantly reduced with a more pronounced effect by ISAM-M89A (figure 2A). To confirm this, antagonist effects of the cell growth, the kinetics of the observed cytotoxic effect mediated by the antagonists was studied using real-time imaging within the same spheroid culture setting (figure 2B). With the low and middle antagonist concentrations being statistically equal in cell viability, the latter concentration of ISAM–140, ISAM–R56A and ISAM–M89A significantly affected the spheroid growth over time compared with untreated control (figure 2C). Despite the low CD3 T cell frequency in these tumor resections (online supplemental figure 4F), blockage of A_2B_AR still reduced cell viability and spheroid growth compared with untreated spheroids.

### Blockade of A_2B_AR rescues adenosine-mediated suppression of T and NK cell proliferation

To elucidate if the antitumor effects observed by the A_2B_AR antagonists could alter lymphocyte activity, the different antagonists were added directly to T and NK cells in the presence of exogenous adenosine. While no statistical difference in viability at different adenosine concentrations was observed, a trend of decreasing viability with increasing adenosine concentration among CD8 T cell subsets was observed. Conversely, the viability of CD56 positive NK cells was not affected by adenosine (online supplemental figure 3B). However, a dose-dependent adenosine-mediated suppression of cell proliferation was observed across the different subtypes of T and NK cells with naïve CD8 T cells being the most affected (online supplemental figure 3C,D).

**Figure 3:**
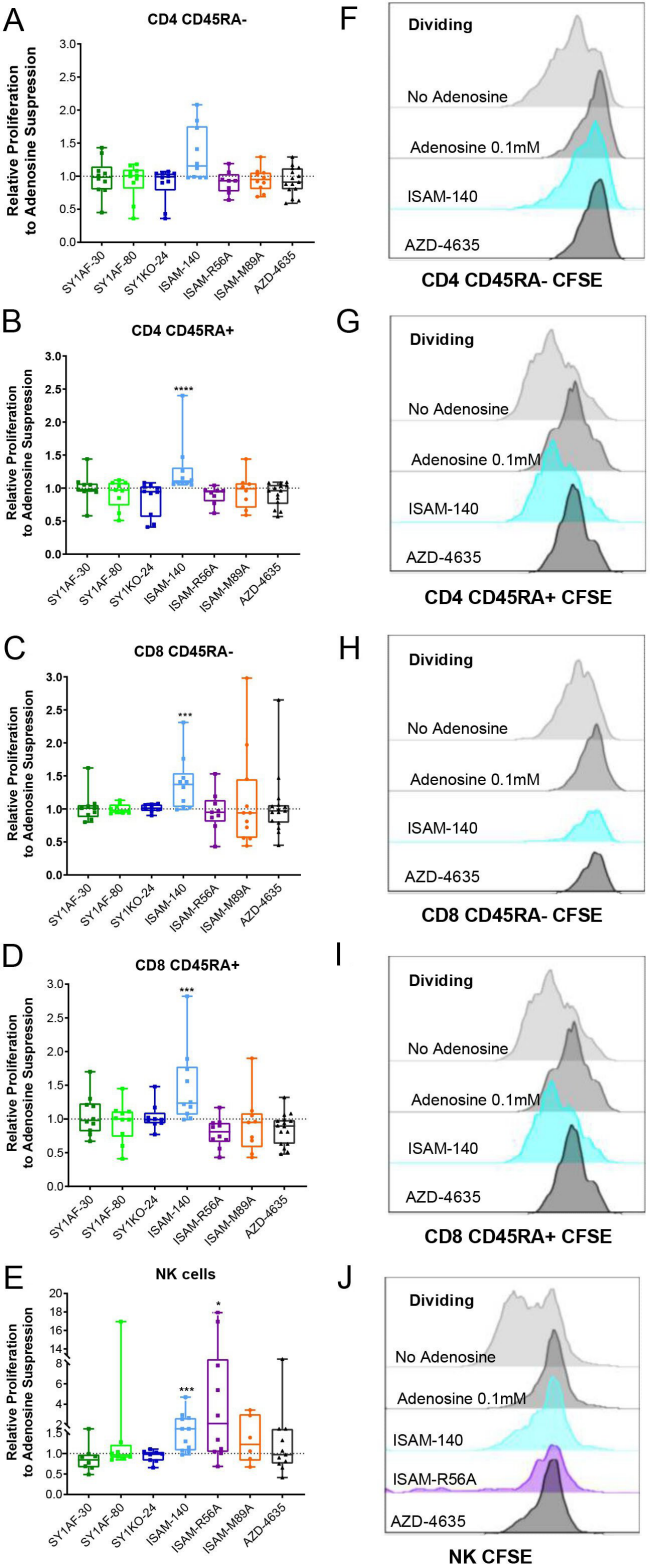
Rescue of lymphocyte proliferation by of A_2B_AR antagonism. Relative proliferation of: (A) CD45RA- CD4 T cells, (B) CD45RA+ CD4 T cells, (C) CD45RA- CD8 T cells, (D) CD45RA+ CD8 T cells, and (E) NK cells, after incubation with exogenous adenosine 0.1 mM and A_2B_AR antagonist 12 µM for 3 (A–D) and 6 (E) days. (n=10 healthy donors) CD45RA expression differentiates naïve and effector T cells from central and effector memory T cells. Box plots with minimum, first quartile, median, third quartile, and maximum are presented. Unpaired and non-parametric statistical analysis—Mann-Whitney test was performed against untreated control with *p<0.05, **p<0.01, ***p<0.001, and ****p<0.0001. (F–J) Representative CFSE histogram on the corresponding lymphocyte subset. CFSE, carboxyfluorescein succinimidyl ester.

Due to its suppressive effect on cell proliferation without compromising viability, adenosine concentration at 0.1 mM was used to investigate the ability of the different A_2B_AR antagonists in rescuing adenosine-mediated suppression of lymphocyte proliferation. A subapoptotic concentration of 12 μM A_2B_AR antagonists was added to proliferating lymphocyte cultures (data not shown). ISAM–140 rescued the proliferation of CD45RA+ (naïve and effector) CD8 T cells, CD45RA- CD4 T cells (central and effector memory) and CD45RA+ CD4 T cells, and NK cells (figure 3). While ISAM–R56A did not restore the proliferation of T cell subsets, NK cell proliferation was significantly rescued. None of the other A_2B_AR antagonists (SY1AF–30, SY1AF–80 and SY1KO–24) had any measurable impact on the rescue of lymphocyte proliferation (figure 3). Similarly, the dual A_2A_AR/A_2B_AR antagonist ISAM–M89A or the A_2A_AR antagonist AZD–4635 did not restore the proliferation of either T or NK cells.

Given the different effects on proliferation across lymphocyte subsets, we hypothesized that the expression of A_2A_AR and A_2B_AR might differ accordingly. Flow cytometry analysis showed a highly donor-dependent variability of the expression intensity of A_2A_AR and A_2B_AR, and no significant difference in their expression was observed between the different lymphocyte populations (online supplemental figure 5). Similarly, the frequency of cells expressing A_2A_AR and A_2B_AR ranging between 10% and 20% did not differ between lymphocyte populations and was highly donor dependent (online supplemental figure 5E,F).

### A_2B_AR antagonists inhibit extracellular ADO by downregulating pAMPK in T cells with upregulation of expression of CD69, IFNy, and perforin

**Figure 4:**
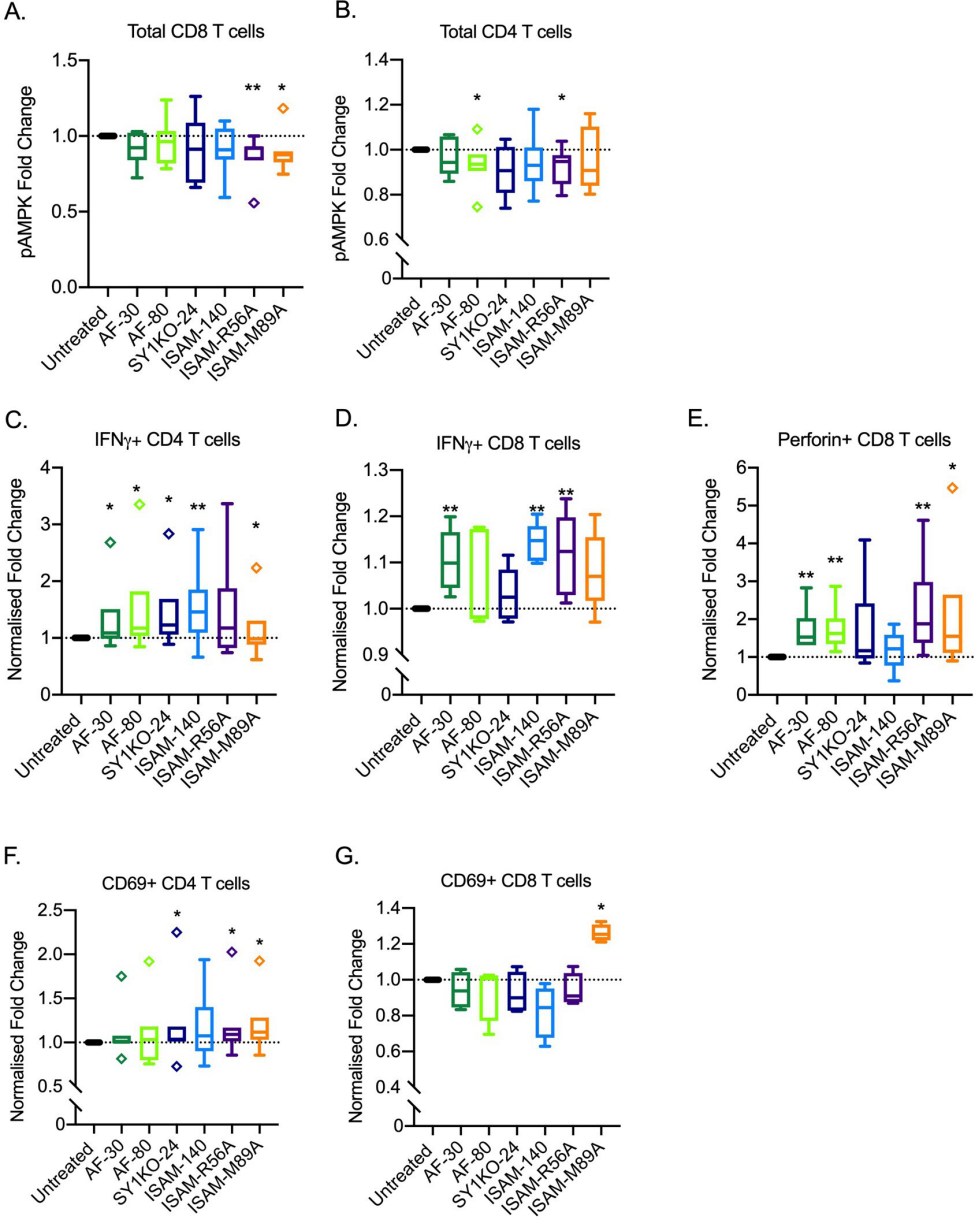
Rescue of lymphocyte proinflammatory cytokine production IFNy and perforin by A_2B_AR antagonism. (A,B) Inhibition of extracellular adenosine uptake via phosphorylated-AMP activated protein kinase (pAMPK) staining (n=7 healthy donors). Production of proinflammatory cytokines—(C,D) IFNy and (E) perforin in CD4 (n=5 healthy donors) and CD8 (n=6 healthy donors) T cells. Expression of CD69 adhesion molecule in (F). CD4 (n=7 healthy donors) and (G) CD8 (n=4 healthy donors) T cells. A_2B_AR antagonist 12 µM was added. Outlier is presented as diamond shape and defined by Tukey’s rule, which is not included in statistical analysis. Box plots with minimum, first quartile, median, third quartile, and maximum are presented. Normalized fold change is based on percent values compared with untreated controls. Unpaired and non-parametric statistical analysis—Mann-Whitney test was performed against untreated control with *p<0.05, **p<0.01, ***p<0.001, and ****p<0.0001.

To demonstrate the ability of A_2B_AR antagonists to inhibit extracellular ADO uptake in lymphocytes, one of the downstream cellular ADO signaling molecules—phosphorylated-AMP activated protein kinase (pAMPK) was analyzed in total CD4 and CD8 T cells (figure 4A,B). ISAM-R56A significantly downregulated pAMPK among both CD4 and CD8 T cells in the presence of exogenous ADO. Similarly, SY1AF-80 and ISAM-M89A significantly inhibited ADO uptake in CD4 and CD8 T cells, respectively.

With the rescue of adenosine-mediated suppression of lymphocyte proliferation, effector function through proinflammatory cytokines—IFNy and perforin, and the early activation marker—CD69 were successively examined. IFNy production by CD4 and CD8 T cells was successfully rescued by several antagonists (figure 4C,D). Similarly, perforin production by cytotoxic CD8 T cells was significantly upregulated on A_2B_AR antagonisms (figure 4E), of which, SY1AF-30 and ISAM-R56A significantly improved both IFNy and perforin production on adenosine suppression. CD69 expression, associated with early lymphocyte activation, was also increased (figure 4F,G) whereby ISAM-M89A had positive effect on both CD4 and CD8 T cells. Finally, both ISAM-R56A and ISAM-M89A rescued killing of breast cancer TILs against autologous tumor cells while ISAM-R56A also rescued killing in sarcoma TILs (online supplemental figure 6).

### A_2B_AR antagonists rescue CD8 naïve T cell proliferation in adenosine-producing tumor spheroids

**Figure 5:**
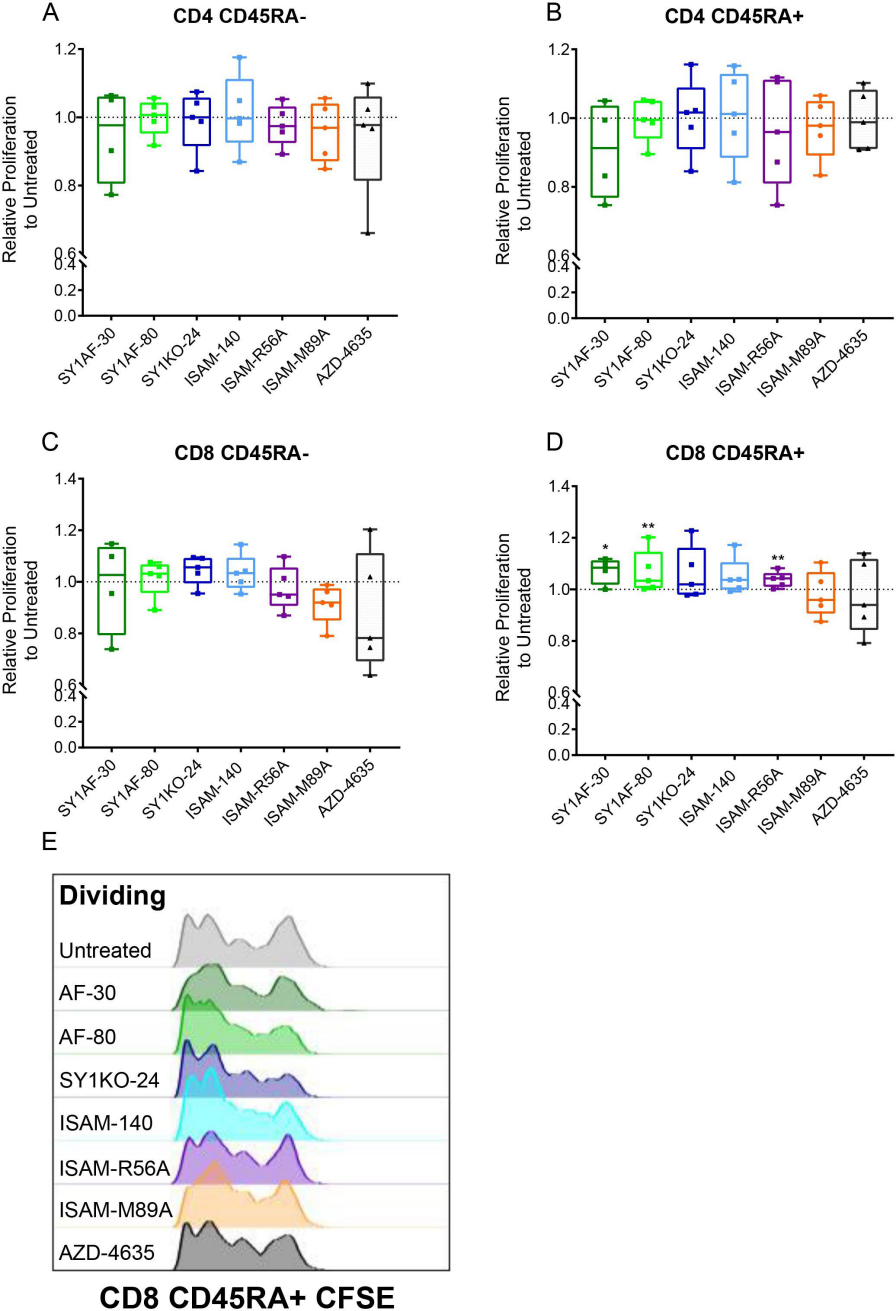
Rescue of lymphocyte proliferation in sarcoma spheroids by A_2B_AR antagonism. Relative proliferation of: (A) CD45RA- CD4 T cells, (B) CD45RA+CD4 T cells, (C) CD45RA- CD8 T cells and (D) CD45RA+ CD8 T cells after antagonist 12 µM treatment for 3 days in MPNST patient-derived sarcoma spheroid. (n=5 healthy donors) Box plots with minimum, first quartile, median, third quartile, and maximum are presented. Unpaired and non-parametric statistical analysis—Mann-Whitney test was performed against untreated control with *p<0.05, **p<0.01, ***p<0.001, and ****p<0.0001. (E) Representative CSFE dilution of CD45RA+ CD8 T cells. CD45RA expression differentiates naïve and effector T cells from central and effector memory T cells. MPNST, malignant peripheral nerve sheath tumors; UPS, undifferentiated pleomorphic sarcoma.

Sarcomas had the highest alteration frequencies for ADORA2B gene amplification as well as highest median copy number at DNA level when compared against 33 TCGA tumor types (online supplemental figure 4C,D). To investigate the effect of A_2B_AR antagonism on lymphocyte proliferation in a more physiological relevant model, a sarcoma spheroid model that produce endogenous adenosine to better mimic the TME instead of using exogenous adenosine was developed. The relative production of adenosine was validated in spheroids including patient-derived tumors and the commercial U2OS cell line (online supplemental figure 7A). The expression of the ectonucleotidases CD39 and CD73 was highest in primary tumor spheroids from undifferentiated pleomorphic sarcoma, myxofibrosarcoma, and malignant peripheral nerve sheath tumors (MPNSTs, online supplemental figure 7B). Based on these results, lymphocyte proliferation was analyzed in MPNST-derived sarcoma spheroids. In contrast to the rescue of lymphocyte proliferation observed with exogenous adenosine, the A_2B_AR antagonists SY1AF–30, SY1AF–80 and ISAM–R56A significantly rescued naïve and effector CD8 T cell proliferation based on CD45RA expression (figure 5).

### A_2B_AR antagonism improves TIL infiltration into autologous patient-derived sarcoma spheroids

**Figure 6:**
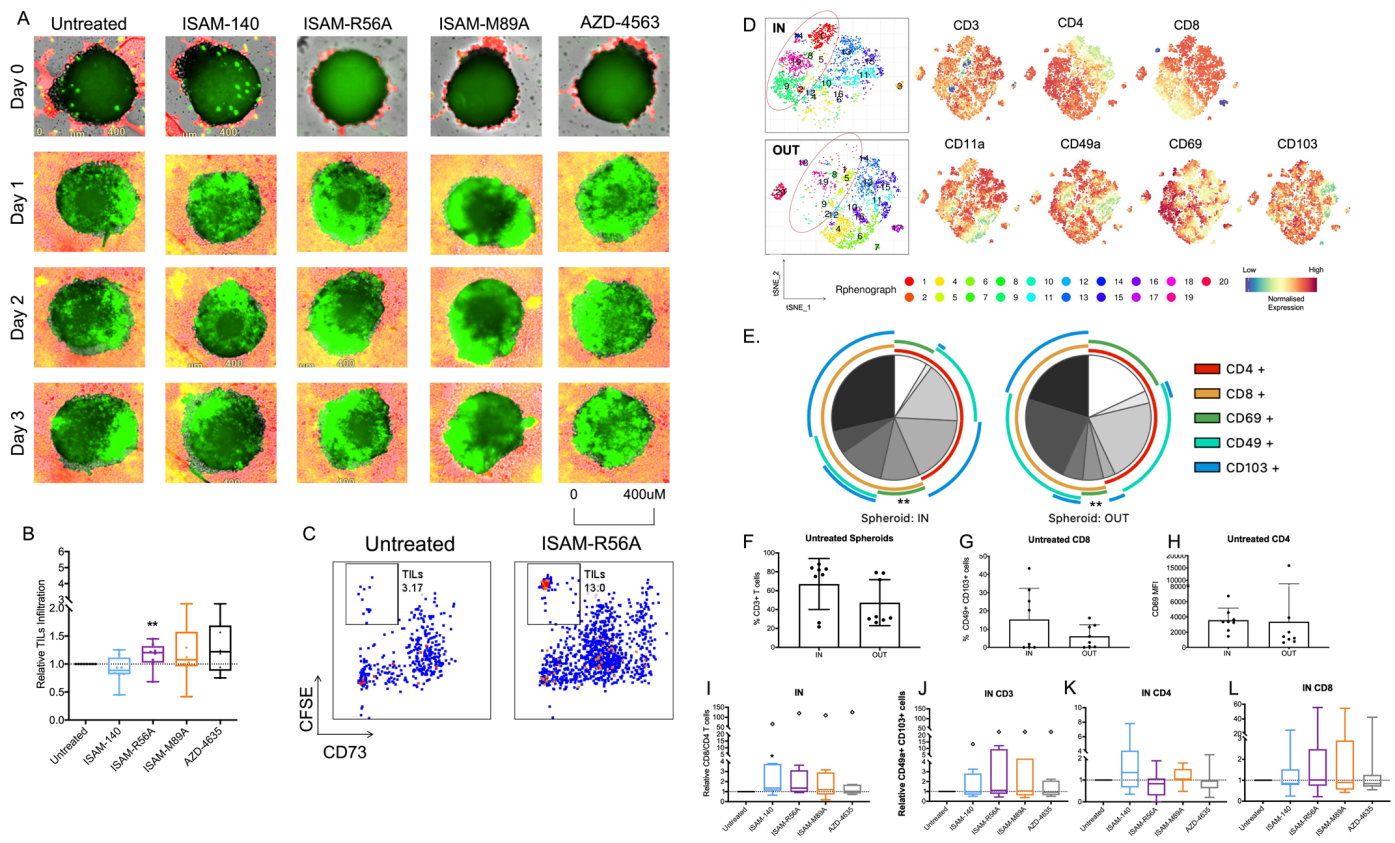
Infiltration of TILs into autologous patient-derived sarcoma spheroids after A_2B_AR antagonist treatment. (A) Representative real-time imaging of spheroid TIL infiltration over 3 days under 4× objective. Green areas demarcate CFSE-labeled TILs infiltrated into the red boundary tumor spheroid. (B) Relative TIL infiltration into autologous spheroids (n=8 donors) measured by flow cytometer. Antagonist 12 µM was added with CSFE-labeled TILs after spheroid formation on day five. (C) Representative flow cytometry plot of CFSE positive TILs in CD73+ tumor spheroid. (D) tSNE analysis and (E) annotated pie chart of TILs within (IN) and outside of (OUT) the spheroids without treatment (n=8 donors, indicated within the pie chart). Wilcoxon Rank Sum test was performed to compare the various IN vs OUT adhesion markers. Expression of (F) CD3 T cells, (G) CD49a+ CD103+ among CD8 T cells and (H) CD69 MFI among CD4 T cells IN and OUT of the spheroids without treatment (n=8 donors). (I) Relative CD8/CD4 TIL ratio IN the spheroids. Differential expression of relative (J–L). CD49a+ CD103+ cells IN the spheroids (n=8 donors) of CD3, CD8, and CD4 TILs. Antagonist 12 µM was added for J–L. Outlier is presented as diamond shape and defined by Tukey’s rule, which is not included in statistical analysis. Box plots with minimum, first quartile, median, third quartile, and maximum are presented. Unpaired and non-parametric statistical analysis—Mann-Whitney test was performed against untreated control with *p<0.05, **p<0.01, ***p<0.001, and ****p<0.0001. CFSE, carboxyfluorescein succinimidyl ester; TIL, tumor infiltrating lymphocyte; tSNE, T-distributed stochastic neighboring embedding.

To increase the translational impact of the immunomodulatory effects of the A_2B_AR antagonists, their ability in rescuing adenosine-mediated suppression was tested in patient-derived ex vivo expanded tumor-infiltrating lymphocytes (TILs) and autologous sarcoma spheroids. These expanded TILs were mainly composed of CD4 and CD8 effector memory cells (online supplemental figure 7C–F). Real-time imaging showed the blockade of A_2B_AR, especially by ISAM-R56A, resulted in an overall improved TIL infiltration in comparison with untreated tumor spheroids (figure 6A,B). Although not statistically significant, both ISAM–R89A and AZD-4635 treatments resulted in a median increased infiltration of 7% and 20%, respectively, whereas ISAM–140 did not have any effect on TIL infiltration (figure 6B). To confirm the frequency of TIL infiltration, the spheroids were collected and analyzed for CSFE positive and CD73 negative TILs by flow cytometry. Despite a high variability, ISAM–R56A treatment showed significantly enhanced TIL infiltration into the spheroids (figure 6C).

With the recruitment and retention of T cells within the tumor, expression of adhesion molecules is required. Thus, the expression of tissue resident T cells markers- CD11a, CD49a, CD69 and CD103, was analyzed to gain insights into the phenotype of spheroid-infiltrated T cells. T-distributed stochastic neighboring embedding (tSNE) analysis revealed that the majority of infiltrating TILs are CD8 positive (cluster 1) and CD4 positive (cluster 9) T cells (figure 6D). Among them, the infiltrated CD8 T cells were enriched with 2.5-fold higher CD49a and CD103 coexpression, while no significant difference was observed, the infiltrated CD4 T cells showed an enrichment of CD69 (figure 6E–H). Furthermore, the total CD3 T cells were 20% greater inside the spheroid. To investigate the effect of A_2B_AR antagonism in modulating the phenotype of infiltrated TILs, various antagonists were added to the TIL-tumor spheroids. All antagonists increased the CD8/CD4 T cell ratio though it was statistically significant only in the presence of ISAM–140 (figure 6I). In contrast, no significant change in the expression of the adhesion molecules CD11a, CD69, and CD49a/CD103 between untreated and A_2B_AR antagonist-treated spheroid cultures was observed (figure 6J–L, online supplemental figure 8).

## Discussion

While blockade of A_2A_AR is well documented to rescue T and NK cell proliferation, the effect of targeting the A_2B_AR in lymphocytes remains almost unexplored.^8 13 16^ Only recently, it was reported that A_2B_AR deficiency in tumor-bearing mice resulted in an increased infiltration of dendritic cells to promote cross-priming of adoptively transferred tumor antigen-specific T cells.^21^ A_2B_AR activation plays critical roles during tumor development including but not limited to proliferation, angiogenesis, invasion and metastasis, as well as immune suppression, which make the antagonists of this receptor hold great promise for the development of new polyvalent cancer therapeutics.^5 6^ To investigate lymphocyte activity on blockade of A_2B_AR, a set of five potent and selective A_2B_AR antagonists and a dual A_2A_AR/A_2B_AR antagonist were synthesized and evaluated. Herein, documented ligands were conceived in the context of a hit to lead program based on the 1,4-dihydrobenzo^4 5^ imidazo[1,2-a]pyrimidine scaffold. The obtained data highlight the potential of this scaffold to provide potent AR antagonists while illustrating how subtle structural modifications can strongly affect the affinity and selectivity profile of the novel ligands. The main SAR and selectivity trends identified within the series were substantiated by a molecular modeling study based on a receptor-driven docking model of A_2B_AR constructed based on the crystal structure specific of the human A_2A_AR.

Given the short half-life of endogenous adenosine,^47^ exogenous adenosine is still commonly used to study adenosine-mediated immunosuppression in the TME. In general, lymphocyte assays are based on phenotypic analysis and often complemented with read-outs to analyze canonical functions such as cell viability, proliferation and the ability to produce inflammatory cytokines as well as to recognize and kill target cells. Throughout these experiments, the A_2B_AR antagonists were evaluated in lymphocyte viability and proliferation assays. Since prognosis is often associated with the frequency of TILs,^51–53^ we developed two models to study the blockage of A_2B_AR in recusing antitumor immune responses. An exogenous adenosine in vitro healthy donor model and endogenously produced adenosine ex vivo patient-derived model were used to better study the dynamic crosstalk of adenosine in the TME as well as being clinically relevance. As a result, A_2B_AR antagonists successfully rescued antitumor immune response through cytotoxicity of patient-derived spheroid cultures, proliferation of lymphocytes and tumor spheroid immune cell infiltration accompanied with differences in phenotype.

The six ligands examined exhibited high A_2B_AR affinity and excellent selectivity profiles. Two of them (ISAM–R56A and ISAM–M89A) were herein originally reported, by introduction of a 2-fluorobenzyl group or a chlorine atom at positions 1 and 8 of the tricyclic core, respectively. Indeed, ISAM–R56A (Ki=1.50 nM) is confirmed as one of the most potent A_2B_AR antagonist published to date, while ISAM–M89A exhibited a highly promising dual A_2B_AR/A_2A_AR antagonistic profile (while devoid of affinity for the remaining AR subtypes). The selected ligands revealed negligible CD73 inhibitory action, thus allowing to attribute the herein observed effects to their specific A_2B_AR antagonistic effect. The high affinity of these compounds could be explained by a computational model of the A_2B_AR in complex with these ligand chemotype, which had been used in the design of these series of antagonists.

By exposure of these A_2B_AR antagonists to breast cancer patient-derived cells, their antitumor activity was first revealed through a reduced tumor spheroids growth rate. Despite the highly variable donor-dependent expression of A_2_ARs, the immunomodulatory effect of A_2B_AR antagonism was further demonstrated with the successful rescue of T and NK cell proliferation under exogenous adenosine-mediated suppression, with CD8 naïve T cell being the most responsive of the T cell types examined. These findings correspond to an adenosine-producing human melanoma cell line, showing higher suppression of CD8 T cell proliferation than CD4 T cells.^54^ A_2B_AR antagonists were subsequently shown to inhibit extracellular ADO uptake via downstream pAMPK in total CD4 and CD8 T cells. Even with varying adenosine susceptibility between donors, improved production of proinflammatory cytokines IFNy and perforin along with the expression of the activation marker CD69 was observed. In addition, the obtained discrepancies could be related with drug availability, binding kinetics and physicochemical properties, influencing a different cell response for drugs with similar affinity.

Patient outcome in various sarcoma types is shown to correlate with the presence of TILs.^52 53 55^ Patient-derived spheroids can retain the unique characteristic of the original tumor compared with 2D monoculture or patient-derived xenograft by enabling cell-cell and cell-extracellular interactions.^56 57^ Using a patient-derived sarcoma spheroids model, we observed that treatment with A_2B_AR antagonists improved autologous TIL infiltration into sarcoma spheroids. Tumor spheroid-infiltrating lymphocytes were enriched for the expression of the tissue resident markers CD49a and CD103 in CD8 T cells and CD69 in CD4 T cells.^58^ A recent study identified a unique population of CD8 TILs coexpressing CD39 and CD103 that were reactive against both primary and metastatic tumors.^59^ Another study defined CD8 tissue resident T cells in human epithelia with cytotoxic function to express CD49a and correlate with inflammatory skin diseases.^60^ In vivo A_2A_AR antagonism has also been reported to upregulate the expression of CD69 on TILs, while A_2B_AR antagonism enhanced CXCR3-dependent TIL responses.^61 62^ Notably, a recent study demonstrated that adenosine can mediate functional and metabolic suppression of tumor-infiltrating CD8+ T cells.^37^ Thereby, A_2B_AR antagonism not only potentially increase TIL infiltration into solid tumors through modulation of adhesion molecules but may also improve the overall metabolic fitness of tumor-infiltrating T cells.

ISAM–140, a potent and highly selective A_2B_AR antagonist (K*_i_* =3.49 nM), was shown as an optimal A_2B_AR binder by computational modeling and proved to be an efficient antagonist in functional cAMP assay.^30^ We here demonstrate that this compound exerts biologically improved immune cell proliferation on T and NK cells in an A_2B_AR expression independent manner in healthy donor PBMCs. This response was dose-dependent with a higher concentration further restoring proliferation (data not shown). This lymphocyte proliferation rescue was accompanied with upregulated IFNy production in both CD4 and CD8 T cells. ISAM–140 was also non-toxic and even improved cell viability (data not shown). In addition, a novel A_2B_AR antagonist—ISAM–R56A, with K*_i_* to the A_2B_AR similar to the corresponding A_2A_AR value of the preclinical A_2A_AR antagonist—AZD–4635 (1.50 nM vs 1.70 nM, respectively), improved TILs infiltration into autologous patient-derived sarcoma spheroids. CD8-naïve T-cell proliferation response was also observed in the adenosine-producing spheroid model, while a specific NK cell proliferation response was noted in the exogenous adenosine assay. CD8 T cell IFNy and perforin production was also significantly rescued under exogenous adenosine suppression. Thereby, the potential of ISAM–R56A to induce tumorous TILs infiltration is highly promising. This induction of tumorous CD8 T cell infiltration was already observed from the clinical trial of a dual A_2A_AR/A_2B_AR antagonist–AB928 combined treatment with anti-PD1.^63^

## Conclusion

With only one A_2B_AR antagonist currently registered in a clinical trial,^3^ our findings close the gap of lacking alternatives and provide insights to practical readouts related to adenosine-mediated immune suppression. We established a systematic workflow to screen novel small molecule antagonists that enabled the identification of ISAM–R56A as the most potent candidate for A_2B_AR blockade. With ISAM–R56A, cytotoxic immune cells can be relieved from adenosine-mediated suppression to proliferate and infiltrate into adenosine-producing solid tumors expressing CD73. Besides using appropriate immunocompetent or xenograft in vivo models to evaluate the pharmacology and preclinical safety of these novel small molecule antagonists as potential complements to existing immunotherapies, future directions on downstream hypoxia-HIF-1a of A_2_AR-cAMP signaling axis shall be mimicked in in vitro and ex vivo assays.

## Data Availability

Data are available on reasonable request.
