# Correction: A2B adenosine receptor antagonists rescue lymphocyte activity in adenosine-producing patient-derived cancer models

**DOI:** 10.1136/jitc-2022-004592corr1

**Published:** 2024-01-25

Tay AHM, Prieto-Díaz R, Neo S, et al. A2B adenosine receptor antagonists rescue lymphocyte activity in adenosine-producing patient-derived cancer models. *J Immunother Cancer* 2022; **10:** e004592. doi: 10.1136/jitc-2022-004592.

In this article The x-axis on figures 4B, D, F and G have been updated to include the correct values.

