# Supplementary material for: A2B adenosine receptor antagonists rescue lymphocyte activity in adenosine-producing patient-derived cancer models
Source: J Immunother Cancer. 2022 May 17;10(5):e004592. doi: 10.1136/jitc-2022-004592 (PMC9115112; doi:10.1136/jitc-2022-004592)
Supplement: Supplementary data [file jitc-2022-004592supp001.pdf]

SUPPORTING INFORMATION

A_{2B} Adenosine Receptor Antagonists Rescue Lymphocyte Activity in Adenosine-Producing Patient-Derived Cancer Models

Apple Tay Hui Min,[1,2,‡] Rubén Prieto-Díaz,[3,‡] Shiyong Neo,[1,4] Le Tong,[1] Xinsong Chen,[1] Valentina, [5] Björn Önfelt,[5,6] Johan Hartman,[1] Felix Haglund,[1] Maria Majellaro,[3] Jhonny Azuaje,[3] Xerardo Garcia-Mera,[3] Jose M. Brea,[6] Maria I. Loza,[6] Willem Jespers,[8] Hugo Gutierrez-de-Teran,[8] Eddy Sotelo,[3,*] and Andreas Lundqvist[1,*]

[1]Department of Oncology-Pathology, Karolinska Institute, Stockholm SE-17177, Sweden. [2]School of Biological Sciences, Nanyang Technological University, Singapore. [3]Center for Research in Biological Chemistry and Molecular Materials (CiQUS), University of Santiago de Compostela, Santiago de Compostela ES-15782, Spain. [4]Singapore Immunology Network SIgN, Agency for Science, Technology and Research, Singapore, Republic of Singapore. [5]Department of Microbiology, Tumor and Cell Biology, Science for Life Laboratory, Karolinska Institutet, Stockholm, Sweden. [6]Department of Applied Physics, Science for Life Laboratory, KTH Royal Institute of Technology, Stockholm SE-11419, Sweden. [7]Center for Research in Molecular Medicine and Chronic Diseases (CiMUS), University of Santiago de Compostela, Santiago de Compostela ES-15782, Spain. [8]Department of Cell and Molecular Biology, Uppsala University, Uppsala SE-75124, Sweden.

Corresponding authors:

*E.S.: phone, +34-881815732; fax, +34 881815704; e-mail, e.sotelo@usc.es.

*A.L.: phone, +46-(0)8-517 768 59; e-mail, andreas.lundqvist@ki.se.

Table of contents

Supplementary methods and results....S2

Molecular dynamics parameters....S3

Chemistry....S4

Synthetic procedures....S5

Supplementary tables....S7

Supplementary figures....S8

Spectroscopic and analytical data....S16

References....S19

Tay AHM, *et al. J Immunother Cancer* 2024; 10:e004592. doi: 10.1136/jitc-2022-004592

Supplementary methods and results

Annexin V viability analysis

Cells were washed with Annexin V binding buffer (BioLegend) then incubated with Alexa Fluor 647 – conjugated Annexin V (BioLegend) at room temperature for 15 minutes. For $A_{2A}AR$ and $A_{2B}AR$ expression profiling, cells were washed with BD Perm/Wash then incubated with BD CytoFix/CytoPerm at 4°C for 20 minutes. The cells were then washed and stained with PE-conjugated $A_{2A}AR$ mouse antibody (Clone 7F6-G5-A2; Santa Cruz Biotechnology, sc-32261 PE) at room temperature for 30 minutes. After washing twice to remove most $A_{2A}AR$ antibody possible, goat $A_{2B}AR$ primary antibody (Clone PA5-18422; Thermo Fisher Scientific) was incubated at 4°C overnight. The cells were subsequently washed and stained with Alexa Fluor 488-conjugated anti-goat secondary antibody (Thermo Fisher Scientific) at room temperature for 1 hour. The cells were then washed with FC buffer twice before acquiring on NovoCyte. $A_{2A}AR$ and $A_{2B}AR$ fluorescence minus one (FMO) staining were also prepared. For differential expression of ADO ectonucleotidases, day five patient-derived sarcoma spheroids were digested and stained for CD45, CD73 and CD39 using the same procedures as cell surface staining (Supplementary Table 1).

Public database bioinformatic analysis

Normalised, batch-corrected, gene expression and DNA copy number of ADORA2B from The Cancer Genome Atlas (TCGA) Pan-Cancer Genome Atlas project (Pan-Can) were accessed and downloaded from USCS Xena Browser (https://xenabrowser.net). Fold change of transcript per million was visualized using Gene Expression Profiling Interactive Analysis (GEPIA) Browser (http://gepia.cancer-pku.cn/). DNA alteration frequency from TCGA Pan-Can were accessed and visualized in cBioPortal (https://www.cbioportal.org).

Chromium (51Cr) release cytotoxicity assay

Chromium (51Cr) release assay was used to measure the autologous TILs-mediated cytotoxicity against the established patient-derived breast and cancer cell lines. Briefly, breast and sarcoma tumor cells were labelled with 51Cr (PerkinElmer) as target cells and seeded on 96-well V-bottom plate (Corning) at 5 000cells/well. Effector TILs were added at the indicated Effector: Target (E:T) ratios. The supernatants were collected carefully into LUMA plates (PerkinElmer) after 24 and 48 hours of co-culture. After drying overnight, radioactivity of the plate was read with MicroBeta2 (PerkinElmer).

Relative adenosine production assay

An ectonucleotidase CD73 assay was optimized to measure the relative ADO production based on competitive AMP blockade and presence of CD73+ cells [47,48]. Day five patient-derived sarcoma spheroids were digested and split into triplicates in X-VIVO20 and 1% PS culture media with an excessive amount of AMP at 0.4mM (Sigma-Aldrich) incubated for 30 minutes. After 1500 RPM was applied for three minutes, 25 μL supernatant were removed and mixed with 25 μL of 200 μM ATP (Sigma-Aldrich) in the same media. 25 μL of this mixture was added to a white opaque OptiPlate (PerkinElmer) containing 25 μL Cell-Titer Glo reagent (Promega, US). Relative luminescence unit (RLU) can be read on SPARK 10M plate reader with an integration time of 100ms.

Molecular dynamics parameters.

A 25Å sphere centered on the center of geometry of the ligand is considered for MD simulations of each generated protein-ligand complex, in order to equilibrate it from the initial docking pose. Protein atoms in the boundary of the sphere (22-25Å outer shell) had a positional restraint of 20 kcal/mol/Å^2, while solvent atoms were subject to polarization and radial restrains using the surface constrained all-atom solvent (SCAAS)[1–3] model to mimic the properties of bulk water at the sphere surface. Atoms lying outside the simulation sphere are tightly constrained (200 kcal/mol/Å^2 force constant) and excluded from the calculation of non-bonded interactions. Long range electrostatics interactions beyond

a 10 Å cut off were treated with the local reaction field method,[4] and all titratable residues outside the sphere were neutralized and histidine residues were assigned a hydrogen atom on the δ nitrogen solvent bond and angles were constrained using the SHAKE algorithm.[5] The OPLS-AA/M force field was used, with compatible ligand parameters generated with the ffld server.[6] The simulation sphere was warmed up from 0.1 to 298 K, during a first equilibration period of 0.61 nanoseconds, where an initial restraint of 25 kcal/mol/Å^2 imposed on all heavy atoms was slowly released for all complexes. Thereafter the system was subject to ten parallel replicates of unrestrained MD, of 0.5 ns length each.

Chemistry

All starting materials, reagents and solvents were purchased and used without further purification. After extraction from aqueous phases, the organic solvents were dried over anhydrous sodium sulfate. The reactions were monitored by thin-layer chromatography (TLC) on 2.5 mm Merck silica gel GF 254 strips, and each purified compound showed a single spot; unless stated otherwise, UV light and/or iodine vapor were used to detect compounds. The reactions were performed in coated Kimble vials on a PLS (6×4) Organic Synthesizer with orbital stirring. Purification of isolated products was carried out by column chromatography (Kieselgel 0.040–0.063 mm, E. Merck).

The purity and identity of all tested compounds were established by a combination of HPLC, high resolution mass spectrometry and NMR spectroscopy. Melting points were determined on a Gallenkamp melting point apparatus and are uncorrected. The NMR spectra were recorded on Bruker AM300 spectrometer. Chemical shifts are given as δ values against tetramethylsilane as internal standard and *J* values are given in Hz. High-resolution mass spectra (HRMS) were obtained on an Autospec Micromass spectrometer. NMR and HRMS reports of novel compounds are shown at the end of Spectroscopical and analytical data section. The unequivocally assignation of the different regioisomers was assisted by NOE experiments. Routinely purity control was performed by analytical HPLC using a Water Breeze™ 2 (binary pump 1525, detector UV/Visible 2489, 7725i Manual Injector Kit 1500 Series) using an Luna® Silica 100 Å, 4.6 mm × 150 mm, 5 µm column with gradient elution using hexane/isopropyl alcohol mixture in different percentages as mobile phase. The purity of all tested compounds was determined to be >95%.

Tay AHM, *et al. J Immunother Cancer* 2024; 10:e004592. doi: 10.1136/jitc-2022-004592

The purity and identity of all tested compounds were established by a combination of HPLC, high resolution mass spectrometry and NMR spectroscopy. Melting points were determined on a Gallenkamp melting point apparatus and are uncorrected. The NMR spectra were recorded on Bruker AM300 spectrometer. Chemical shifts are given as δ values against tetramethylsilane as internal standard and *J* values are given in Hz. A copy of the NMR spectra is found in the supplementary data file. High-resolution mass spectra (HRMS) were obtained on an Autospec Micromass spectrometer. HRMS reports of novel compounds are shown in Supporting information. The unequivocally assignation of the different regioisomers was assisted by NOE experiments. Routinely purity control was performed by analytical HPLC using a Water Breeze™ 2 (binary pump 1525, detector UV/Visible 2489, 7725i Manual Injector Kit 1500 Series) using a Luna® Silica 100 Å, 4.6 mm × 150 mm, 5 µm column with gradient elution using hexane/isopropyl alcohol mixture in different percentages as mobile phase. The purity of all tested compounds was determined to be ≥95%.

Synthetic procedures

Procedure for the Biginelli synthesis of 3,4-dihydropyrimidin-2(1*H*)-ones SY1AF-30[7] and SY1AF-80[7]

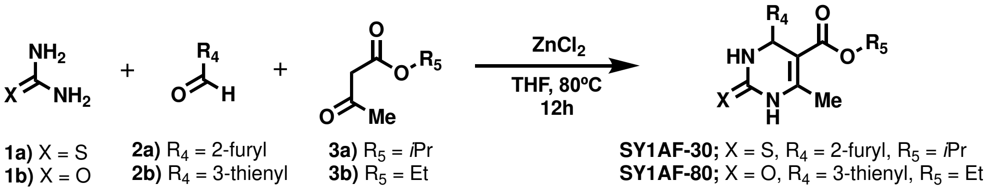

A mixture of the urea **1a** or thiourea **1b** (7.5 mmol), 2-furancarboxaldehide **1a** or 3-thiophenecarboxaldehyde **1b** (5 mmol), isopropyl **3a** or ethyl **3b** acetoacetate (5 mmol) and $ZnCl_2$ (0.5 mmol) in 3 mL of THF in coated Kimble vials was stirred with orbital stirring at 80ºC for 12h. After completion of the reaction, as indicated by TLC, the reaction mixture was poured onto crushed ice and stirred for 5-10 minutes. The solid separated was filtered under suction, washed with ice-cold water (20 mL), and then purified either by recrystallization or column chromatography on silica gel.

Tay AHM, *et al. J Immunother Cancer* 2024; 10:e004592. doi: 10.1136/jitc-2022-004592

Procedure for the Biginelli synthesis of isopropyl 2-(cyanoimino)-4-(furan-2-yl)-6-methyl-1,2,3,4-tetrahydropyrimidine-5-carboxylate SY1KO-24[8]

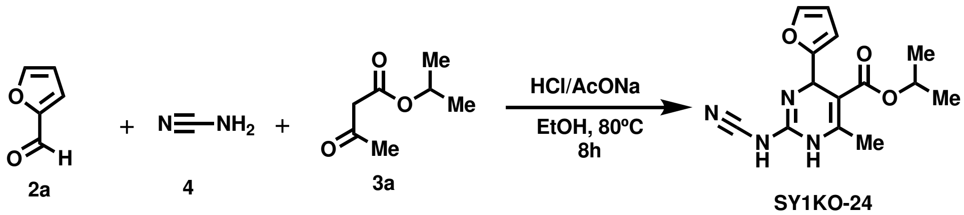

A mixture of cyanamide **4** (2 mmol), 2-furanecarboxaldehyde **2a** (1 mmol), isopropyl acetoacetate **3a**, sodium acetate (1 mmol), and concentered hydrochloric acid (0.5 mL) in 7 mL of ethanol in a coated Kimble vial was stirred by orbital stirring at 80ºC for 8h. After completion of the reaction, as indicated by TLC, the reaction mixture was poured onto crushed ice and stirred for 10 min. The solid was filtered under suction, washed with ice-cold water (20 mL) and then purified either by recrystallization or column chromatography on silica gel.

Procedure for the Biginelli synthesis of isopropyl 4-(furan-2-yl)-2-methyl-1,4-dihydrobenzo[4,5]imidazo[1,2-a]pyrimidine-3-carboxylate ISAM-140[9]

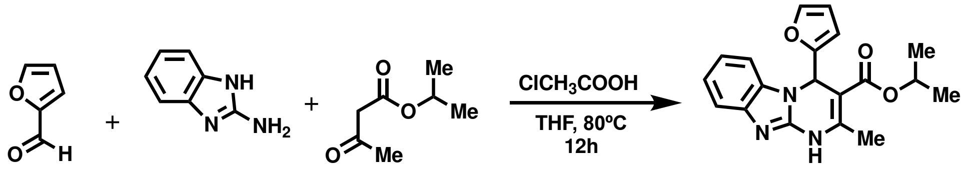

A mixture of 2-aminobenzimidazole **5** (7.5 mmol), 2-furanecarboxaldehyde **2a** (5 mmol), isopropyl acetoacetate **3a** (5 mmol), and 2-chloroacetic acid (0.05 mmol) in 3 mL of THF in coated Kimble vials was stirred with orbital stirring at 80ºC for 12h. After completion of the reaction, as indicated by TLC, the reaction mixture was poured onto crushed ice and stirred for 10 min. The solid was filtered under suction, washed with ice-cold water (20 mL) and then purified either by recrystallization or column chromatography on silica gel.

Tay AHM, *et al. J Immunother Cancer* 2024; 10:e004592. doi: 10.1136/jitc-2022-004592

Supplementary tables

Supplementary Table 1. List of cell surface antibodies used in flow cytometry. A. $A_{2A}AR$ & $A_{2B}AR$ expression profiling **B.** CFSE Proliferation and Annexin V viability assay **C.** ADO ectonucleotidase expression **D.** Spheroid infiltrated TIL phenotyping **E**. Extracellular ADO uptake assay. **F**. IFNy and Perforin cytokine production assay **G.** Breast tumor resection phenotyping.

Antigen	Conjugate	Distributor	Catalog Number	A	B	C	D	E	F	G
CD3 (T cell)	PerCP/Cy5.5	BioLengend	30429	✓			✓			
	PE-CF594	BD Bioscience	562280		✓			✓	✓	
	Brilliant Violet 650	BD Bioscience	563852							✓
CD4 (T helper cell)	Pacific Blue	BD Bioscience	558116	✓	✓		✓	✓	✓	
	PE	BD Bioscience	555347							✓
CD8 (Cytotoxic T cell)	PE/Cy7	eBioscience	25-0088-42	✓	✓		✓	✓	✓	✓
CD45RA (Naïve T cell)	Brilliant Violet 650	BioLegend	304136	✓	✓					
CD27 (Central memory marker)	Brilliant Violet 605	BioLegend	302830	✓	✓					
CD56 (NK cell)	APC/Cy7	BioLegend	318331	✓						
	Brilliant Violet 570	BD Horizon	564058		✓		✓			
		BioLegend	318330							✓
CD19 (B cell)	Brilliant Violet 570	BioLegend	302235	✓						
CD45 (Leukocyte)	Brilliant Violet 650	BioLegend	304044			✓				
	PerCP-eFluro 710	eBioscience	46-0459-42							✓
CD73	APC/Cy7	BioLegend	344022			✓				
CD39	PE/Cy7	BioLegend	328211			✓				
CD11a	PE	BioLegend	301208				✓			
CD49a	APC/Fire 750	BioLegend	328317				✓			
CD69	Brilliant Violet 785	BioLegend	310931				✓		✓	
CD103	APC	BioLegend	350215				✓			
IFNy	eFluor450	eBioscience	48-7319-42						✓	
Perforin	PE	BioLegend	308106						✓	
CD11b	APC-Cy7	BD Bioscience	557754							✓
CD11c	PE-Cy7	BD Bioscience	561356							✓
CD14	Brilliant Violet 785	BD Bioscience	563698							✓
CD15	Alexa Fluor 488	BioLegend	301910							✓
CD68	PE-CF594	BD Bioscience	564944							✓
HLA-DR	Brilliant Violet 650	BD Bioscience	564231							✓
CD90	APC	BD Bioscience	559869							✓
CD31	Brilliant Violet 421	BD Bioscience	564089							✓
EPCAM	Brilliant Violet 785	BioLegend	324238							✓
Live/Dead marker	Aqua	Thermo Fisher Scientific	L34966	✓		✓	✓			✓
	Near-IR	Thermo Fisher Scientific	L10119		✓			✓	✓	

Supplementary figures

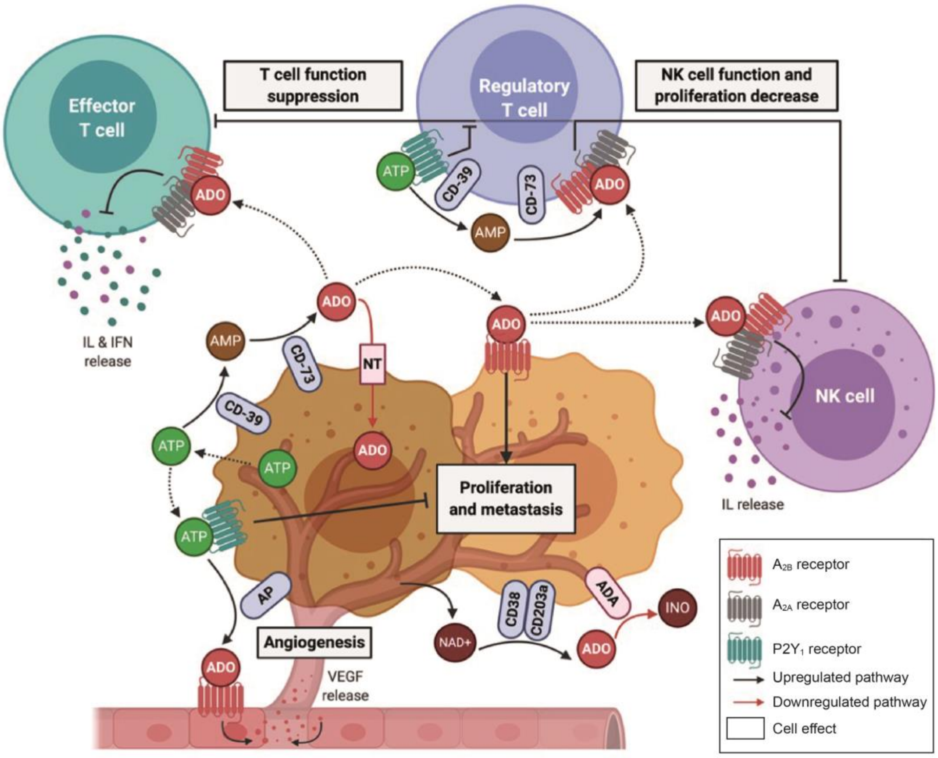

Supplementary Figure 1. Adenosine-mediated effects in the tumor microenvironment (TME). Hypoxia induces the release of ATP and NAD+ leading to an increase in the extracellular adenosine (ADO) concentration. The accumulated ATP can either activate P2 purinergic receptors or it can be metabolized by the consecutive action of ectonucleotidases CD39 and CD73 to ADO. Furthermore, NAD+ can be transformed to ADO by the CD38/CD203a enzymatic system. The increased ADO concentration is also a consequence of the fact that both ADO reuptake by nucleoside transporters (NT) and adenosine deaminase (ADA) metabolization are downregulated in tumor cells. In the vascular endothelium, ADO interacts with A2BAR, triggering the release of pro-angiogenic factors like VEGF. Activation of A2BAR promotes tumor growth and metastasis by increasing cell proliferation, inhibiting apoptosis and reducing cell adhesion. Activation of both A2A and A2B ARs by ADO also results in immunosuppressive patterns, downregulating inflammatory cytokine secretion, as well as decreasing the effector function and proliferation of T and natural killer (NK) cells. This figure was created with Biorender.com.

Tay AHM, *et al. J Immunother Cancer* 2024; 10:e004592. doi: 10.1136/jitc-2022-004592

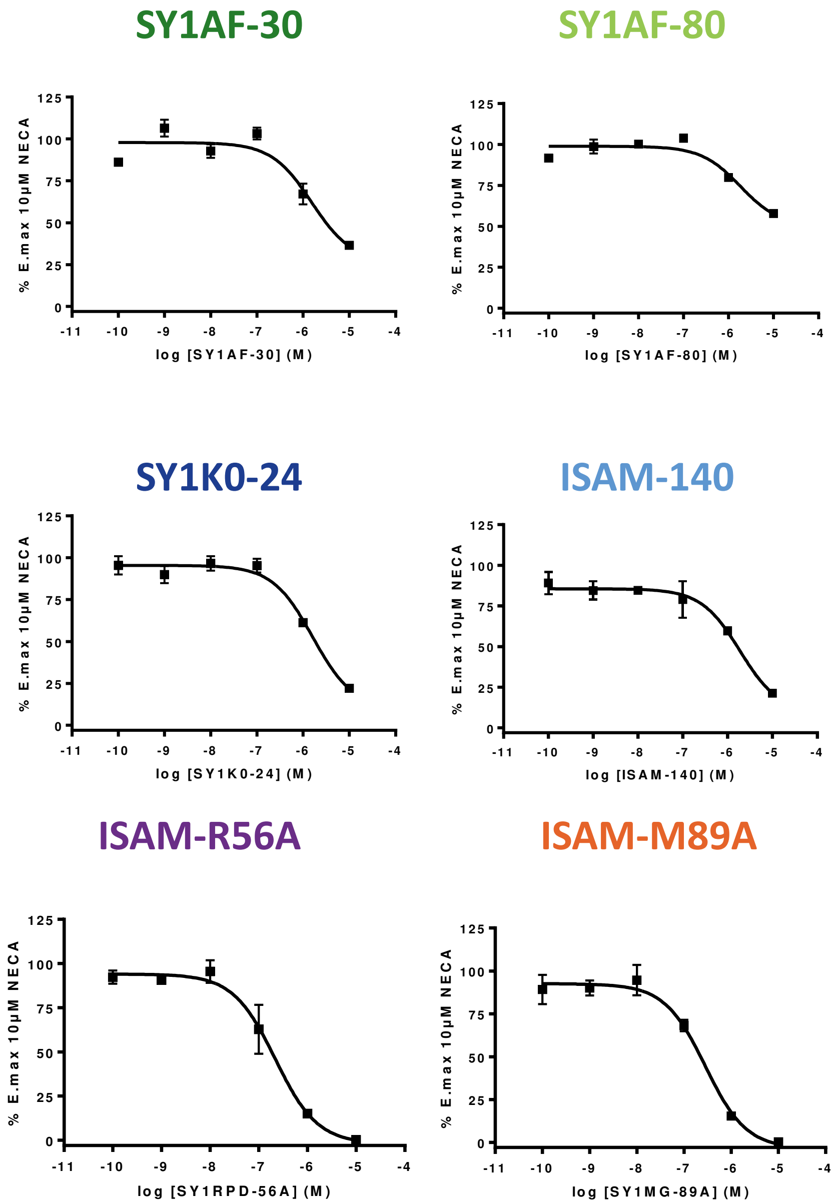

Supplementary Figure 2. Concentration-response curves for cAMP production assay using NECA and test compound.

Tay AHM, *et al. J Immunother Cancer* 2024; 10:e004592. doi: 10.1136/jitc-2022-004592

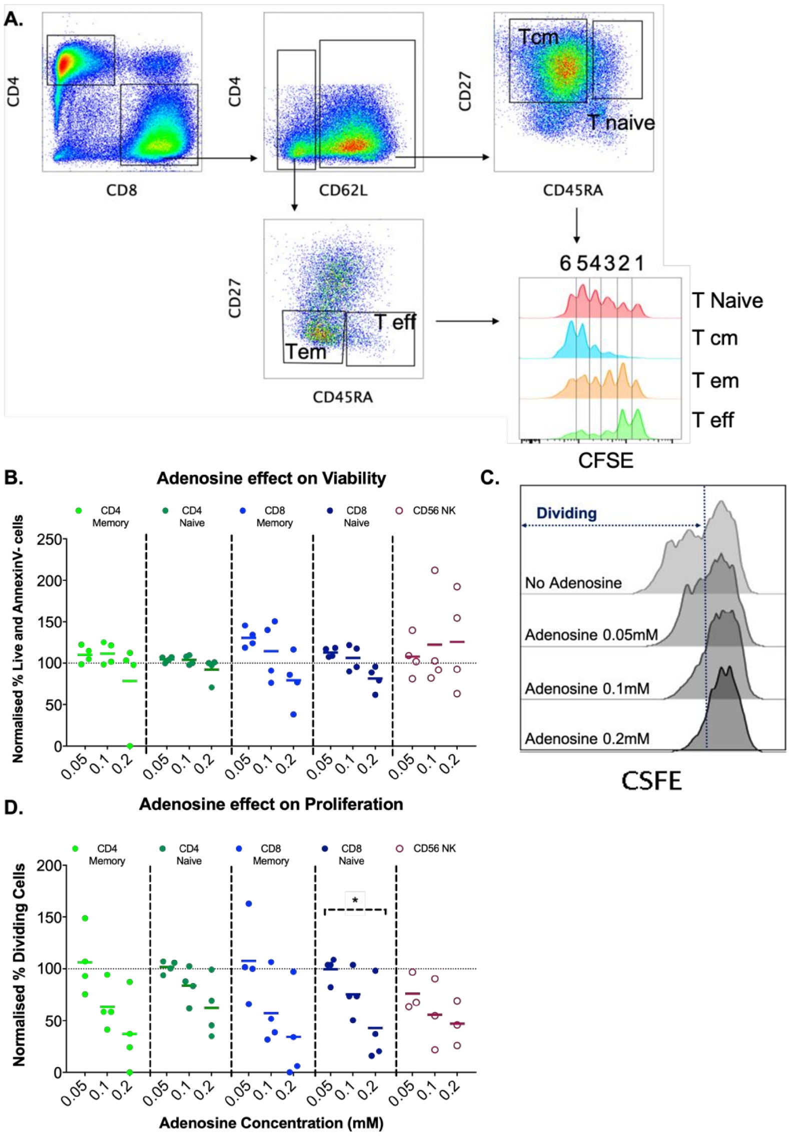

Supplementary Figure 3. Adenosine effects on viability and proliferation in healthy donor peripheral blood T and NK cells. **A**. Representative gating strategies for T cell subsets analyzed by flow cytometry. **B**. Adenosine effect on viability for CD3 T cell subsets and CD56 NK cells after 3-day and 6-day incubation respectively. **C**. Representative CSFE histogram on naïve CD8 T cells dividing cells. **D**. Adenosine effect on proliferation for CD3 T cell subsets and CD56 NK cells after 3-day and 6-day incubation respectively. Normalized percentage to no adenosine suppression (B and D). Mean from 4 different healthy donors are presented. Statistical analysis – 1-way ANOVA (within each cell type) is performed with * $p<0.05$, ** $p<0.01$, *** $p<0.001$ and **** $p<0.0001$. No statistical significance is not stated. Tcm: Central memory, Tem: Effector memory, Teff: Effector

Tay AHM, *et al. J Immunother Cancer* 2024; 10:e004592. doi: 10.1136/jitc-2022-004592

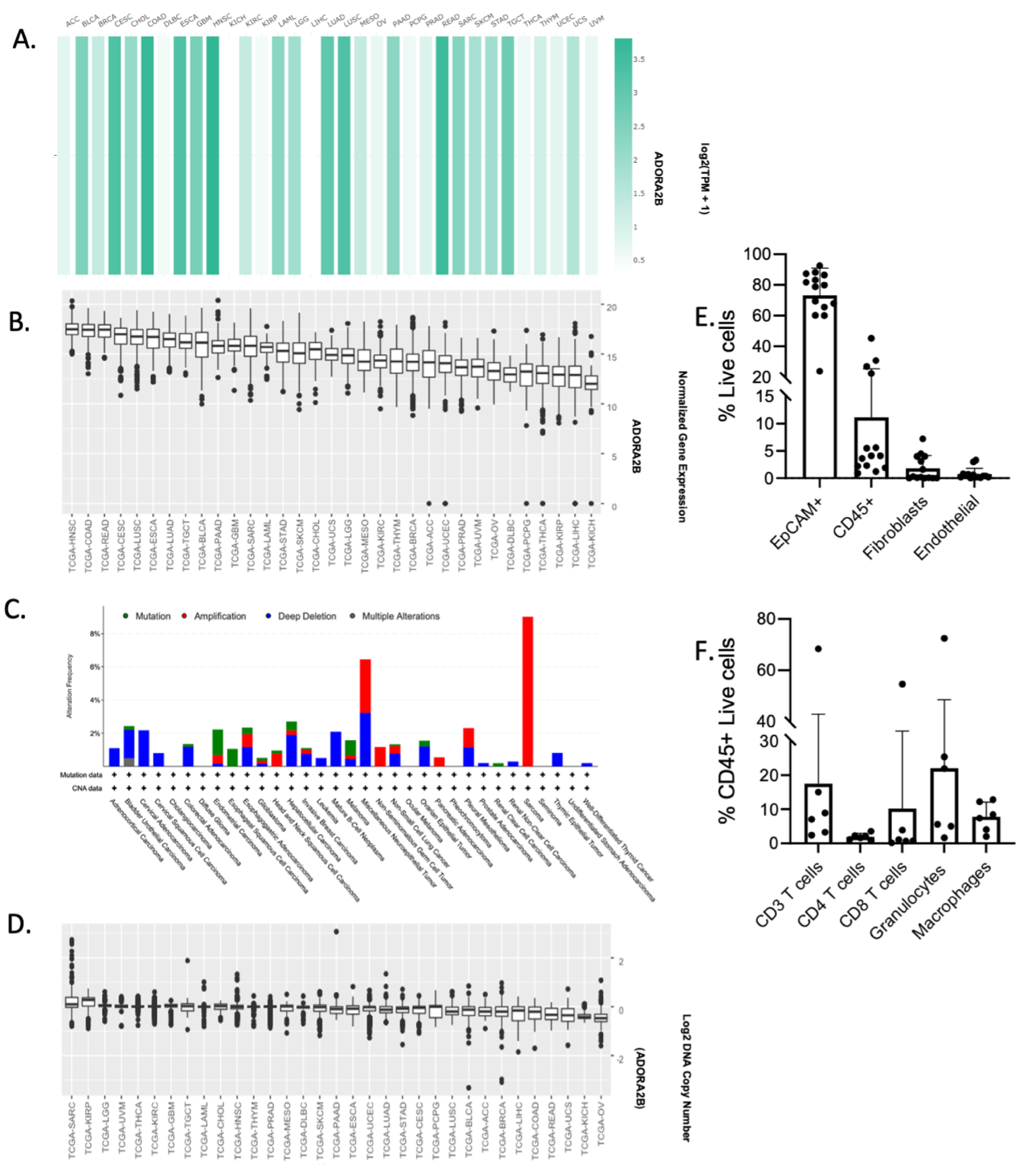

Supplementary Figure 4. TCGA – Pan cancer analysis across 33 tumor types of ADORA2B **A.** Log2 Fold Change, **B.** Normalized Gene Expression, **C.** Alteration Frequency, and **D.** Log2 DNA Copy Number. Composition of breast cancer **E.** tumor resections (n=12 tumors) and **F.** CD45+ immune cells (n=6 tumors).

Tay AHM, *et al. J Immunother Cancer* 2024; 10:e004592. doi: 10.1136/jitc-2022-004592

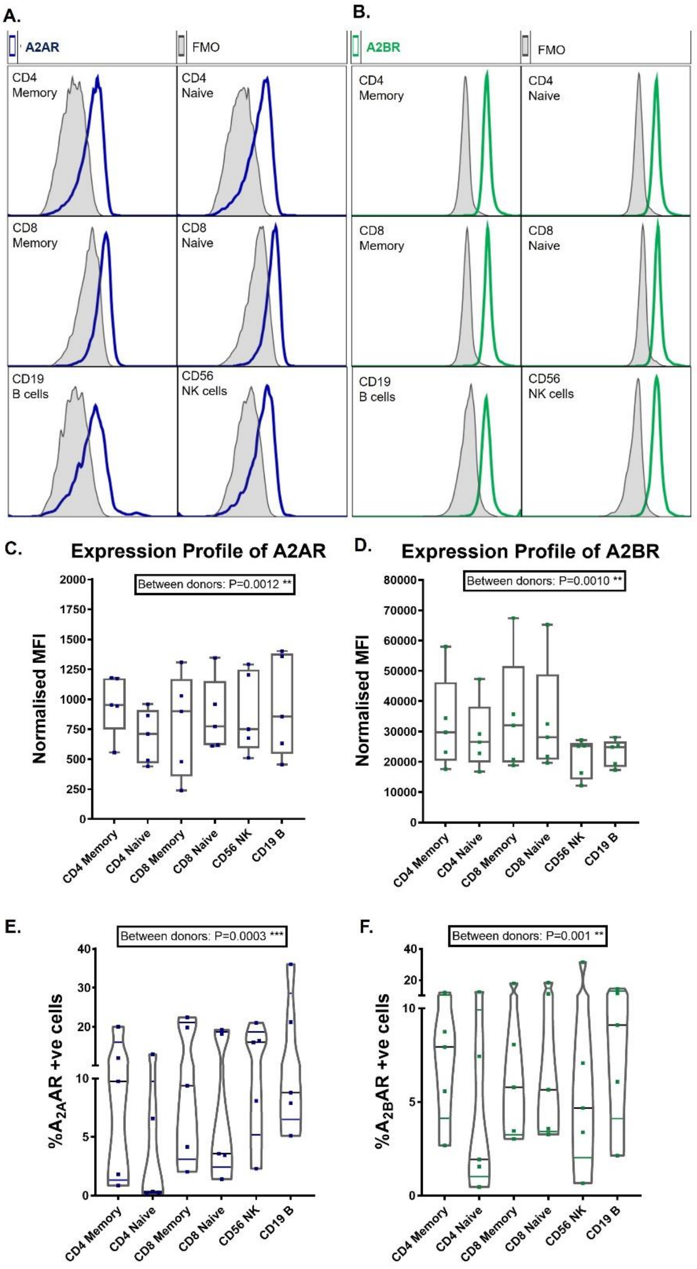

Supplementary Figure 5. Expression profile of $A_{2A}AR$ and $A_{2B}AR$ in healthy donor lymphocyte subsets. Representative histogram of **A**. $A_{2A}AR$ and **B**. $A_{2B}AR$. Filled histograms show Fluorescence Minus One (FMO) control staining. Expression of **C and E**. $A_{2A}AR$ and **D and F**. $A_{2B}AR$ from healthy donors (n=5). Box plots with minimum, first quartile, median, third quartile, and maximum are presented. Statistical analysis – 1-way ANOVA is performed with * $p<0.05$, ** $p<0.01$, *** $p<0.001$ and **** $p<0.0001$.

Tay AHM, *et al. J Immunother Cancer* 2024; 10:e004592. doi: 10.1136/jitc-2022-004592

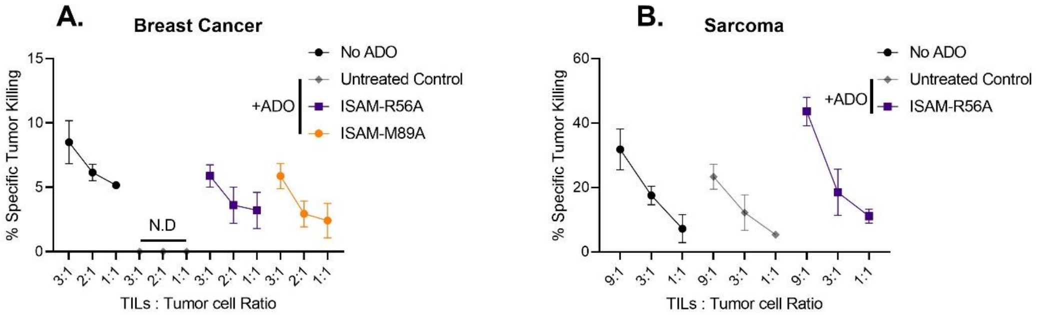

Supplementary Figure 6. A. Breast cancer and **B.** Sarcoma TIL-mediated cytotoxicity of autologous tumor cells in the presence or absence of 0.1M synthetic ADO for 48 and 24 hours respectively. ADO: Adenosine, N.D: No difference, E:T ratio: Effector TILs: Target Cancer cells ratio.

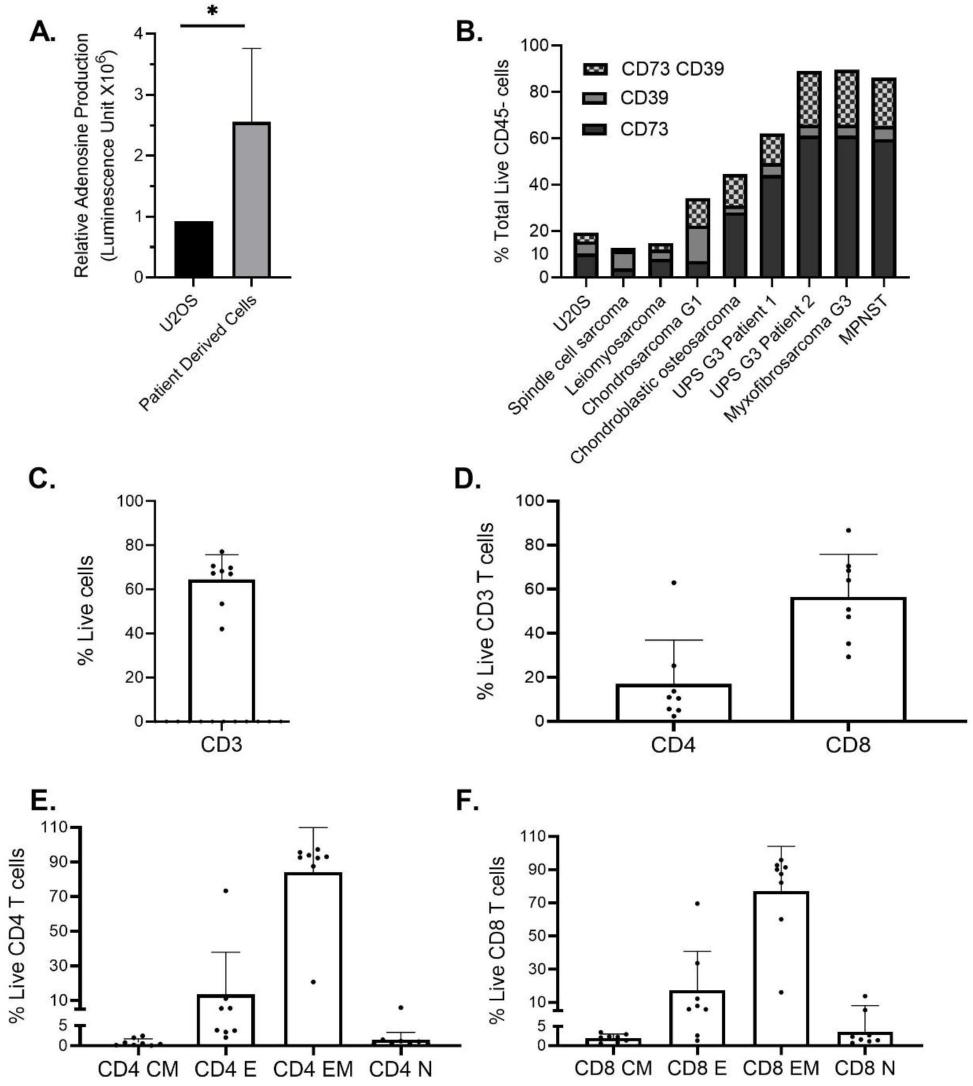

Supplementary Figure 7. A. Relative adenosine production measured by ectonucleotidase CD73 assay and **B**. differential expression of CD73 and CD39 ectonucleotidases measured by flow cytometry of patient-derived sarcoma spheroids (n=8) and the commercial U2OS 3D culture. Composition of expanded TILs (n=8). Expression of **C**. CD3 **D**. CD4 and CD8 **E**. CD4 T cell subsets and **F**. CD8 T cell subsets. MPNST: malignant peripheral nerve sheath tumors. UPS: Undifferentiated pleomorphic sarcoma. CM: Central Memory, E: Effector, EM: Effector Memory, N: Naïve.

Tay AHM, *et al. J Immunother Cancer* 2024; 10:e004592. doi: 10.1136/jitc-2022-004592

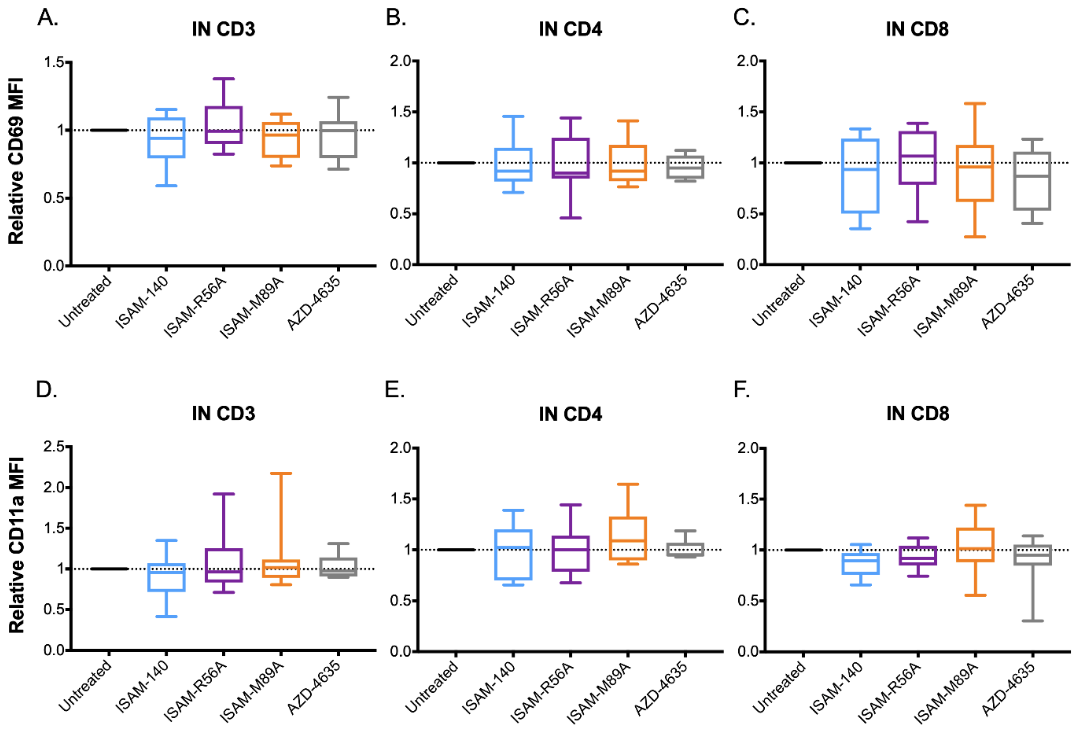

Supplementary Figure 8. Differential expression of relative **A – C.** CD69 and **D – F.** CD11a MFI IN the spheroids (n=8) of CD3, CD8 and CD4 TILs. Antagonist 0.012mM is added.

Spectroscopic and analytical data

(±) Isopropyl 4-(furan-2-yl)-6-methyl-2-thioxo-1,2,3,4-tetrahydropyrimidine-5-carboxylate (SY1AF-30).[7] Yield: 68%. M.p.: 195-196 ºC. ^{1}H-NMR ($CDCl_3$, 300 MHz), δ (ppm): 7.87 (br s, 1H), 7.33 (d, *J*=1.7 Hz, 1H), 7.22 (br s, 1H), 6.29 (dd, *J*=1.9 Hz, *J*=3.2 Hz, 1H), 6.16 (d, *J*=3.2 Hz, 1H), 5.48 (d, *J*=3.1 Hz, 1H), 5.06-4.99 (m, 1H), 2.37 (s, 3H), 1.23 (d, *J*=6.3 Hz, 3H), 1.12 (t, *J*=6.3 Hz, 3H). ^{13}C-NMR (DMSO-d_6, 75 MHz), δ (ppm): 165.4, 153.5, 149.1, 144.2, 139.3, 129.7, 109.8, 99.9, 67.1, 46.6, 22.5, 22.3, 18.3. Elem. Anal. calculated for $C_{13}H_{16}N_2O_3S$, C, 55.70; H, 5.75; N, 9.99; found C, 55.74; H, 5.78; N, 10.02.

(±) Ethyl 6-methyl-2-oxo-4-(thiophen-3-yl)-1,2,3,4-tetrahydropyrimidine-5carboxylate (SY1AF-80).[7] Yield: 88%. M.p.: 232-233 ºC. ^{1}HNMR (DMSO-$d_{6,}$ 300 MHz), δ (ppm): 9.17 (br s, 1H), 7.74 (br s, 1H), 7.44 (dd, *J*=3.0 Hz, *J*=5.0 Hz, 1H), 7.13 (d, *J*=2.8 Hz, 1H), 6.98 (dd, =1.2 Hz, *J*=5.0 Hz, 1H), 5.20 (d, *J*=3.4 Hz, 1H), 4.04 (c, *J*=7.0 Hz, 2H), 2.21 (s, 3H), 1.14 (t, *J*=7.0 Hz, 3H). ^{13}C-NMR (DMSO-d_6, 75 MHz), δ (ppm): 165.5, 152.8, 148.6, 145.9, 126.8, 126.3, 20.9, 99.6, 59.4, 49.6, 17.9, 14.3. Elem. Anal. calculated for $C_{12}H_{14}N_2O_3S$, C, 54.12; H, 5.30; N, 10.52; found C, 54.15; H, 5.29; N, 11.53.

(±) isopropyl 2-(cyanoimino)-4-(furan-2-yl)-6-methyl-1,2,3,4-tetrahydropyrimidine-5-carboxylate (SY1KO-24).[8] Yield 82%. M.p.: 212-214 ºC. ^{1}H NMR (300 MHz, DMSO-d_6) δ (ppm): 10.16 (bs, 1H), 9.09 (bs, 1H), 7.58 (d, *J* = 2.1 Hz, 1H), 6.38 (t, *J* = 2.2, 1H), 6.16 (d, *J* = 3.3 Hz, 1H), 5.29 (d, *J* = 3.9 Hz, 1H), 4.87 (p, *J* = 6.2 Hz, 1H), 2.26 (s, 3H), 1.17 (d, *J* = 6.1 Hz, 3H), 1.07 (d, *J* = 6.0 Hz, 3H). ^{13}C NMR (75 MHz, DMSO-d_6) δ (ppm): 164.3, 155.7, 154.8, 147.2, 143.2, 116.7, 111.0, 106.9, 99.6, 67.5, 47.7, 22.1, 21.9, 17.7. HRMS (EI) m/z: calcd for $C_{14}H_{16}N_4O_3$ $[M]^+$: 288.1222; found: 288.1225.

(±) Isopropyl 4-(furan-2-yl)-2-methyl-1,4-dihydrobenzo[4,5]imidazo[1,2-*a*]pyrimidine-3-carboxylate (ISAM-140).[9] Yield 61%. M.p.: 253−255 °C. ^{1}H NMR (300 MHz, DMSO-d_6), δ (ppm): 10.78 (brs, 1H), 7.67−7.23 (m, 3H), 7.19−6.84 (m, 2H), 6.52 (s, 1H), 6.44 (d, *J* = 3.3 Hz, 1H), 6.37−6.23 (m, 1H), 4.86 (h, *J* = 6.3 Hz, 1H), 2.44 (s, 3H), 1.21 (d, *J* = 6.2 Hz, 3H), 1.05 (d, *J* = 6.1 Hz, 3H). ^{13}C NMR (75 MHz, DMSO-d_6), δ (ppm): 165.0, 153.3, 148.0, 146.0, 143.0, 142.6, 132.0, 122.3, 120.7,

Tay AHM, *et al. J Immunother Cancer* 2024; 10:e004592. doi: 10.1136/jitc-2022-004592

117.2, 110.8, 110.2, 108.2, 94.9, 67.0, 49.7, 22.3, 22.0, 19.1. HRMS (ESI) m/z: calcd for $C_{19}H_{20}N_3O_3$ $[M + H]^+$: 338.1488; found: 338.7927.

(±) Isopropyl 1-(2-fluorobenzyl)-4-(furan-2-yl)-2-methyl-1,4-dihydrobenzo[4,5]-imidazo-[1,2-a]pyrimidine-3-carboxylate (ISAM–R56A). Yield 27%. Mp: 182-183 ºC. ^{1}H NMR (300 MHz, $CDCl_3$) δ (ppm): 7.72 – 7.51 (m, 1H, Ar-H), 7.52 (dd, *J* = 7.3, 2.0 Hz, 1H, Ar-H), 7.41 – 7.00 (m, 7H, Ar-H), 6.61 (s, CH), 6.31 (d, *J* = 1.7 Hz, 2H, Ar-H), 5.82 (d, *J* = 17.4 Hz, 1H, $CH_2C_6H_4F$), 5.52 (d, *J* = 17.4 Hz, 1H, $CH_2C_6H_4F$), 5.13 (hept, *J* = 6.3 Hz, 1H, $OCH(CH_3)_2$), 2.64 (s, 3H, CH_3), 1.31 (d, *J* = 6.2 Hz, 3H, $OCH(CH_3)_2$), 1.21 (d, *J* = 6.26 Hz, 3H, $OCH(CH_3)_2$). ^{13}C NMR (75 MHz, $CDCl_3$) δ (ppm): 165.4, 160.5 (d, *J* = 246.3 Hz), 153.0, 148.3, 147.5, 142.2, 141.8, 132.9, 129.1 (d, *J* = 8.1 Hz, Ar-C), 127.8 (d, *J* = 3.6 Hz), 124.6 (d, *J* = 3.6 Hz), 124.3 (d, *J* = 13.7 Hz), 122.6, 121.2, 117.7, 115.5 (d, *J* = 21.1 Hz), 110.3, 109.5, 107.6, 98.9, 67.8, 49.5, 42.5, 22.1, 21.7, 15.8. HRMS (APCI) *m/z* calcd. for $C_{26}H_{25}FN_3O_3$ $[M+H]^+$: 446.1874; found: 446.1876.

(±) Isopropyl 10-(2-fluorobenzyl)-4-(furan-2-yl)-2-methyl-1,4-dihydrobenzo[4,5]-imi-dazo[1,2-a]pyrimidine-3-carboxylate (ISAM–R56B). Yield: 38%. Mp: 166-167 ºC. ^{1}H NMR (300 MHz, $CDCl_3$) δ (ppm): 7.42 – 7.21 (m, 4H, Ar-H), 7.12 (m, 5H, Ar-H), 6.61 (s, 1H, CH), 6.43 – 6.21 (m, 2H, Ar-H), 5.52 – 5.31 (m, 2H, $CH_2C_6H_4F$), 5.12 (hept, *J* = 6.4 Hz, 1H, $OCH(CH_3)_2$), 2.61 (s, 3H, CH_3), 1.3 (d, *J* = 6.3 Hz, 3H, $OCH(CH_3)_2$), 1.21 (d, *J* = 6.3 Hz, 3H, $OCH(CH_3)_2$). ^{13}C NMR (75 MHz, $CDCl_3$) δ (ppm): 166.3, 160.5 (d, *J* = 246.3 Hz), 160.2, 154.9, 148.9, 141.8, 130.6, 130.1 (d, *J* = 3.7 Hz), 129.7 (d, *J* = 8.2 Hz), 129.2, 124.6 (d, *J* = 3.6 Hz), 122.7 (d, *J* = 42.7 Hz), 115.5 (d, *J* = 21.5 Hz), 110.4, 109.7, 108.8 (d, *J* = 2.8 Hz), 107.7, 95.9, 66.4, 50.4, 38.5 (d, *J* = 5.3 Hz), 24.9, 22.3, 22.0. HRMS (APCI) *m/z* calcd. for $C_{26}H_{25}FN_3O_3$ $[M+H]^+$: 446.1874; found: 446.1877.

(±) Isopropyl 8-chloro-4-(furan-3-yl)-2-methyl-1,4-dihydrobenzo[4,5]imidazo[1,2-a]pyrimidine-3-carboxylate (ISAM-M89A). Yield: 37%. Mp 256 – 258 ºC. ^{1}H NMR (300 MHz, $CDCl_3$), *δ* (ppm): 11.95 (brs, 1H, NH), 7.48 (s, 1H, Ar-H), 7.38 (s, 1H, Ar-H), 7.25 (s, 1H, Ar-H), 7.06-7.21 (m, 2H, Ar-H), 6.52 (s, 1H, CH), 6.11 (s, 1H, Ar-H), 5.11-5.05 (m, 1H, $OCH(CH_3)_2$), 2.70 (s, 3H, CH_3), 1.35 (d, *J* = 6.1 Hz, 3H, $OCH(CH_3)_2$), 1.31 (d, *J* = 6.0 Hz, 3H, $OCH(CH_3)_2$). ^{13}C NMR (75 MHz, $CDCl_3$), *δ* (ppm): 165.3, 147.8, 145.6, 142.3, 141.7, 130.3, 128.2, 126.4, 126.1, 122.7, 121.3, 116.3,

110.2, 99.4, 67.8, 52.1, 21.9, 19.5. HRMS (APCI) *m/z* calcd. for $C_{19}H_{19}ClN_3O_3$ $[M+H]^+$: 372.1109; found: 372.1111.

(±) Isopropyl 7-chloro-4-(furan-3-yl)-2-methyl-1,4-dihydrobenzo[4,5]imidazo[1,2-a]pyrimidine-3-carboxylate (ISAM-M89B). Yield: 35%. Mp 241 – 243 ºC. ^{1}H NMR (300 MHz, $CDCl_3$), δ (ppm): 7.38 (m, 2H, Ar-H), 7.06-7.28 (m, 3H, Ar-H), 6.42 (s, 1H, CH), 6.13 (m, 1H, Ar-H), 5.14-4.99 (m, 1H, OC*H*(CH_3)$_2$), 2.65 (s, 3H, CH_3), 1.37 (d, *J* = 6.2 Hz, 3H, OCH(C*H*$_3$)$_2$), 1.26 (d, *J* = 6.2 Hz, 3H, OCH(C*H*$_3$)$_2$). ^{13}C NMR (75 MHz, $CDCl_3$), δ (ppm): 165.1, 147.6, 146.0, 143.6, 139.8, 139.7, 132.1, 126.6, 125.3, 123.1, 117.0, 109.7, 108.7, 98.8, 67.8, 48.4, 22.2, 21.9, 19.3. HRMS (APCI) *m/z* calcd. for $C_{19}H_{19}ClN_3O_3$ $[M+H]^+$: 372.1109; found: 372.1111.

References

(1) King, G., and Warshel, A. (1989) A surface constrained all-atom solvent model for effective simulations of polar solutions. *J. Chem. Phys. 91*, 3647. DOI: 10.1063/1.456845.

(2) Marelius, J., Kolmodin, K., Feierberg, I., Åqvist, J., and Aqvist, J. (1998) Q: a molecular dynamics program for free energy calculations and empirical valence bond simulations in biomolecular systems. *J. Mol. Graph. Model. 16*, 213–225. DOI: 10.1016/S1093-3263(98)80006-5.

(3) Mastelic-Gavillet, B., Navarro Rodrigo, B., Décombaz, L., Wang, H., Ercolano, G., Ahmed, R., Lozano, L. E., Ianaro, A., Derré, L., Valerio, M., Tawadros, T., Jichlinski, P., Nguyen-Ngoc, T., Speiser, D. E., Verdeil, G., Gestermann, N., Dormond, O., Kandalaft, L., Coukos, G., Jandus, C., Ménétrier-Caux, C., Caux, C., Ho, P. C., Romero, P., Harari, A., and Vigano, S. (2019) Adenosine mediates functional and metabolic suppression of peripheral and tumor-infiltrating CD8+ T cells. *J. Immunother. Cancer 7*, 1–16. DOI: 10.1186/s40425-019-0719-5.

(4) Lee, F. S., and Warshel, A. (1992) A local reaction field method for fast evaluation of long-range electrostatic interactions in molecular simulations. *J. Chem. Phys. 97*, 3100–3107. DOI: 10.1063/1.462997.

(5) Ryckaert, J.-P. J., Ciccotti, G., and Berendsen, H. J. . H. (1977) Numerical integration of the cartesian equations of motion of a system with constraints: molecular dynamics of n-alkanes. *J. Comput. Phys. 23*, 327–341. DOI: 10.1016/0021-9991(77)90098-5.

(6) Kaminski, G. A.; Friesner, R. A.; Tirado-Rives, J.; Jorgensen, W. L. (2001) Evaluation and reparametrization of the OPLS-AA force field for proteins via comparison with accurate quantum chemical calculations on peptides. *J. Phys. Chem. 105*, 6474–6487. DOI:

10.1021/jp003919d.

(7) Crespo, A., Maatougui, A. El, Biagini, P., Azuaje, J., Coelho, A., Brea, J., Garc, X., and Gutie, H. (2013) Discovery of 3,4-Dihydropyrimidin-2(1H)-ones As a Novel Class of Potent and Selective A2B Adenosine Receptor Antagonists. *ACS Med. Chem. Lett. 2*, 1031–1036. DOI: 10.1021/ml400185v.

(8) Carbajales, C., Azuaje, J., Oliveira, A., Loza, M. I., Brea, J., Cadavid, M. I., Masaguer, C. F., García-Mera, X., Gutiérrez-De-Terán, H., and Sotelo, E. (2017) Enantiospecific Recognition at the A2B Adenosine Receptor by Alkyl 2-Cyanoimino-4-substituted-6-methyl-1,2,3,4-tetrahydropyrimidine-5-carboxylates. *J. Med. Chem. 60*, 3372–3382. DOI: 10.1021/acs.jmedchem.7b00138.

(9) El Maatougui, A., Azuaje, J., González-Gómez, M., Miguez, G., Crespo, A., Carbajales, C., Escalante, L., García-Mera, X., Gutiérrez-De-Terán, H., and Sotelo, E. (2016) Discovery of Potent and Highly Selective A2B Adenosine Receptor Antagonist Chemotypes. *J. Med. Chem. 59*, 1967–1983. DOI: 10.1021/acs.jmedchem.5b01586.
