# Supplementary material for: A2B adenosine receptor antagonists rescue lymphocyte activity in adenosine-producing patient-derived cancer models
Source: J Immunother Cancer. 2022 May 17;10(5):e004592. doi: 10.1136/jitc-2022-004592 (PMC9115112; doi:10.1136/jitc-2022-004592)

A_{2B} Adenosine Receptor Antagonists Rescue Lymphocyte Activity in Adenosine-Producing Patient-Derived Cancer Models

Authors

Apple Tay Hui Min, Rubén Prieto-Díaz, Shiyong Neo, Le Tong, Xinsong Chen, Valentina Carannante, Björn Önfelt, Johan Hartman, Felix Haglund, Maria Majellaro, Jhonny Azuaje, Xerardo Garcia-Mera, Jose M. Brea, Maria I. Loza, Willem Jespers, Hugo Gutierrez-de-Teran, Eddy Sotelo and Andreas Lundqvist

Correspondence

e.sotelo@usc.es
andreas.lundqvist@ki.se

In Brief

A_{2B} Adenosine Receptor (A_{2B}AR) and dual A_{2A}/A_{2B} Adenosine Receptor antagonists rescue T and NK cell proliferation, cytokine production and reduced the tumor growth enhanced by adenosine (ADO) in ADO-producing patient derived cancer models.

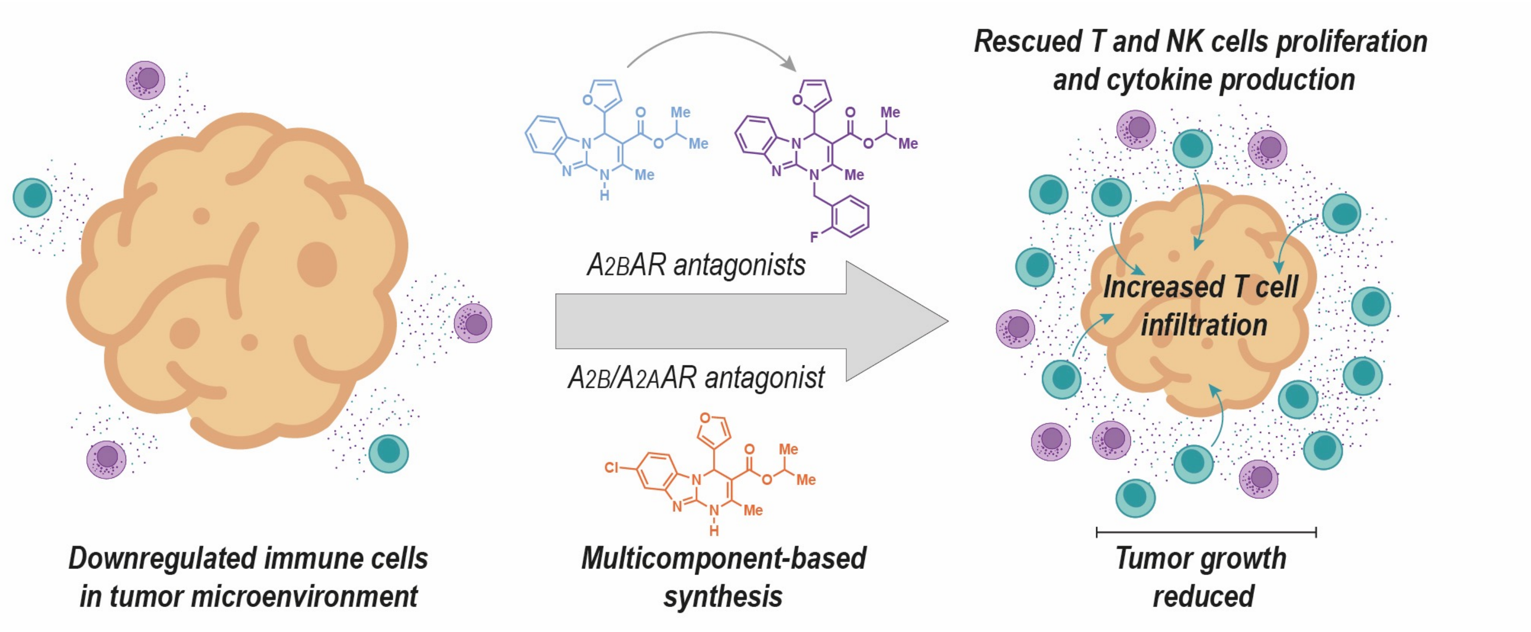

Supplement: Supplementary data [file jitc-2022-004592supp002.pdf]
